# Mathematical and Physical Description of Transport Phenomena in Heat Pipes Based on Nanofluids: A Review

**DOI:** 10.3390/nano15100757

**Published:** 2025-05-18

**Authors:** Marina S. Astanina, Nikita S. Gibanov, Igor V. Miroshnichenko, Egor A. Tarasov, Mikhail A. Sheremet

**Affiliations:** Laboratory on Convective Heat and Mass Transfer, Tomsk State University, 634050 Tomsk, Russia; astanina.marina@bk.ru (M.S.A.); fire9n@mail.ru (N.S.G.); miroshnichenko@mail.tsu.ru (I.V.M.); eatarasov1987@gmail.com (E.A.T.)

**Keywords:** heat pipe, nanofluids, heat transfer, thermal performance, numerical models

## Abstract

Heat pipes are highly efficient heat transfer devices relying on phase-change mechanisms, with performance heavily influenced by working fluids and operational dynamics. This review article comprehensively examines hydrodynamics and heat transfer in heat pipes, contrasting conventional working fluids with nanofluid-enhanced systems. In the present work we discuss mathematical models governing fluid flow and heat transfer, emphasizing continuum and porous media approaches for wick structures. Functional dependencies of thermophysical properties (e.g., viscosity, surface tension, thermal conductivity) are reviewed, highlighting temperature-driven correlations and nanofluid modifications. Transport mechanisms within wicks are analyzed, addressing capillary-driven flow, permeability, and challenges posed by nanoparticle integration. Fourth, interfacial phase-change conditions—evaporation and condensation—are modeled, focusing on kinetic theory and empirical correlations. Also, numerical and experimental results are synthesized to quantify performance enhancements from nanofluids, including thermal resistance reduction and capillary limit extension, while addressing inconsistencies in stability and pressure drop trade-offs. Finally, applications spanning electronics cooling, aero-space, and renewable energy systems are evaluated, underscoring nanofluids’ potential to expand heat pipe usability in extreme environments. The review identifies critical gaps, such as long-term nanoparticle stability and scalability of lab-scale models, while advocating for unified frameworks to optimize nanofluid selection and wick design. This work serves as a foundational reference for researchers and engineers aiming to advance heat pipe technology through nanofluid integration, balancing theoretical rigor with practical feasibility.

## 1. Introduction

Heat pipes, renowned for their exceptional thermal efficiency and passive operation, have become indispensable in applications ranging from electronics cooling to renewable energy systems. Their performance hinges on the working fluid’s ability to transport latent heat through cyclic evaporation and condensation. Conventional fluids, such as water, ethanol, or liquid metals, have been optimized over decades to balance thermophysical properties like thermal conductivity, viscosity, and surface tension. However, as modern technologies demand higher heat fluxes and compact designs, these traditional fluids increasingly face limitations, including capillary limits, thermal resistance bottlenecks, and operational range constraints.

The emergence of nanofluids—base fluids engineered with suspended nanoparticles (e.g., Al_2_O_3_, Cu, CNTs)—has introduced a paradigm shift in heat pipe research. By dispersing nanoparticles (typically 1–100 nm in size) into conventional fluids, nanofluids leverage unique mechanisms such as enhanced thermal conductivity, improved wettability, and modified phase-change dynamics. Studies have demonstrated that nanofluids can increase effective thermal conductivity, reduce thermal resistance, and extend capillary limits through optimized surface interactions. These improvements stem from nanoparticle-induced effects, including Brownian motion, microconvection, and the formation of nanoscale porous layers on heated surfaces. Such advancements position nanofluids as promising candidates for high-performance heat pipes in extreme environments, such as aerospace thermal control or next-generation electronics.

It should be noted that the integration of nanofluids is not without challenges. Increased viscosity due to nanoparticle loading can elevate hydrodynamic resistance, potentially offsetting gains in capillary pumping. Long-term stability issues, such as particle sedimentation, threaten consistent performance and raise practical deployment concerns [1,2]. Furthermore, the complex interplay between nanoparticles and phase-change phenomena, such as altered nucleation rates and interfacial thermal resistance, remains incompletely understood. These trade-offs highlight a critical gap in the literature: a systematic comparison of nanofluid-enhanced heat pipes against their conventional counterparts, weighing benefits against unresolved technical barriers.

This review article addresses this gap by comprehensively analyzing hydrodynamics and thermal transport in heat pipes with and without nanofluids. Investigating experimental and computational studies, we evaluate how nanoparticles reshape key parameters, thermal conductivity, viscosity, surface tension, and phase-change efficiency, and assess their net impact on heat pipe performance. We also explore the role of nanoparticle concentration, material, and size in optimizing thermal-fluidic behavior. Ultimately, this work aims to provide researchers and engineers with a balanced framework for selecting working fluids, guiding future innovations in nanofluid design and heat pipe engineering.

## 2. Mathematical Formulation and Governing Equations

**Figure 1:**
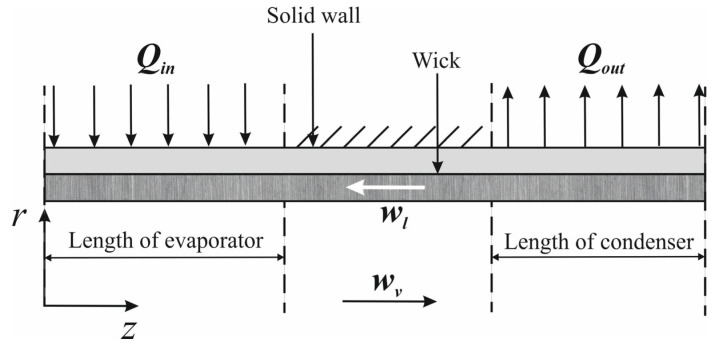
Two-dimensional axisymmetric configuration of a heat pipe.

Mathematical modeling plays a pivotal role in understanding the complex interplay of fluid dynamics, heat transfer, and phase-change phenomena in heat pipes. These models range from simplified analytical frameworks to advanced computational fluid dynamics (CFD) simulations, each tailored to capture specific aspects of heat pipe behavior under varying operational conditions. For conventional heat pipes, the governing equations typically integrate conservation laws for mass, momentum, and energy, coupled with empirical correlations for phase-change processes. The heat pipe consists of three main regions, namely the vapor, the wick structure and the wall region (see Figure 1). The vapor flow can be modeled as 2D, steady, laminar flow with constant viscosity [3,4,5]:

(1) $$\frac{1}{r}\frac{\partial }{\partial r}\left(\rho_{v}rv_{v}\right)+\frac{\partial }{\partial z}\left(\rho_{v}w_{v}\right)=0,$$

(2) $$\frac{1}{r}\frac{\partial }{\partial r}\left(\rho_{v}{rv^{2}}_{v}\right)+\frac{\partial }{\partial z}\left(\rho_{v}v_{v}w_{v}\right)=-\frac{\partial p}{\partial r}+\frac{4}{3}\mu_{v}\frac{1}{r}\frac{\partial }{\partial r}\left(r\frac{\partial v_{v}}{\partial r}\right)+\mu_{v}\frac{\partial^{2}v_{v}}{\partial z^{2}}+\mu_{v}\left(\frac{1}{3}\frac{\partial^{2}w_{v}}{\partial z\partial r}-\frac{4}{3}\frac{v_{v}}{r^{2}}\right),$$

(3) $$\frac{1}{r}\frac{\partial }{\partial r}\left(\rho_{v}rv_{v}w_{v}\right)+\frac{\partial }{\partial z}\left(\rho_{v}{w^{2}}_{v}\right)=-\frac{\partial p}{\partial z}+\frac{\mu_{v}}{r}\frac{\partial }{\partial r}\left(r\frac{\partial w_{v}}{\partial r}\right)+\frac{4}{3}\mu_{v}\frac{\partial^{2}w_{v}}{\partial z^{2}}+\frac{1}{3}\mu_{v}\left(\frac{1}{r}\frac{\partial v_{v}}{\partial z}-\frac{\partial^{2}v_{v}}{\partial z\partial r}\right),$$

(4) $$\rho_{v}c_{p,v}\left(v_{v}\frac{\partial T_{v}}{\partial r}+w_{v}\frac{\partial T_{v}}{\partial z}\right)=k_{v}\left[\frac{1}{r}\frac{\partial }{\partial r}\left(r\frac{\partial T_{v}}{\partial r}\right)+\frac{\partial^{2}T_{v}}{\partial z^{2}}\right]+\frac{Dp_{v}}{Dt}+\mu_{v}\Phi ,$$

(5) $$\Phi =2\left[{\left(\frac{\partial v_{v}}{\partial r}\right)}^{2}+{\left(\frac{v_{v}}{r}\right)}^{2}+{\left(\frac{\partial w_{v}}{\partial z}\right)}^{2}\right]+{\left(\frac{\partial v_{v}}{\partial z}+\frac{\partial w_{v}}{\partial r}\right)}^{2}-\frac{2}{3}{\left[\frac{1}{r}\frac{\partial }{\partial r}\left(rv_{v}\right)+\frac{\partial w_{v}}{\partial z}\right]}^{2}.$$

Here, *r*—radius, *p*—pressure, $v_{v}$—radial velocity, $w_{v}$—axial velocity, $T_{v}$—temperature, $\mu_{v}$—dynamic viscosity, $c_{p,v}$—specific heat, $\rho_{v}$—density, Φ—viscous dissipation. The following abbreviation is used here ν—a vapor.

The equation of state can be written in the following form:

(6) $$p_{v}=\rho_{v}R_{g}T_{v}$$

To describe the process of heat conduction in the solid walls of a heat pipe, the heat conduction equation is often used:

(7) $$\frac{1}{r}\frac{\partial }{\partial r}\left(r\frac{\partial T_{v}}{\partial r}\right)+\frac{\partial^{2}T_{v}}{\partial z^{2}}=0$$

The thermophysical properties of working fluids in heat pipes, particularly nanofluids, are governed by complex functional dependencies that link parameters such as thermal conductivity, viscosity, surface tension, and pressure to variables like temperature, nanoparticle concentration, and material composition. These relationships are critical for modeling heat pipe performance, as they directly influence hydrodynamic behavior and thermal transport efficiency. However, the sheer diversity of proposed correlations, derived experimentally, theoretically, or through hybrid approaches, poses a challenge for systematic analysis. A comprehensive review of all existing models would exceed the scope of this section, but their overarching principles and limitations can be distilled to guide researchers and engineers.

The hydrodynamics and thermal exchange within a wick’s porous matrix arise from intricate multiphase interactions involving the solid wick material, the liquid phase filling its pores, and dispersed nanoparticles. In computational models, nanofluid transport through a mesh-like wick is typically represented as a steady-state, two-dimensional flow through a porous medium. Here, the wick is conceptualized as a rigid, interconnected solid framework with a defined porosity, where the pore network is entirely occupied by the nanoparticle-laden fluid.

A key assumption in such models is the attainment of local thermal equilibrium among the wick, liquid, and nanoparticles. Nevertheless, the incorporation of nanoparticles necessitates adjustments to the thermophysical properties of the base fluid in the governing equations. These adjustments include defining effective thermal conductivity, nanofluid density, and nanofluid viscosity to account for particle-fluid interactions. The liquid motion within the wick is simulated as a steady, two-dimensional flow in porous media with effective properties. Continuity equation, the radial and the axial momentum equation and energy equation can be written in the following form [3,5]:

(8) $$\frac{1}{r}\frac{\partial }{\partial r}\left(rv_{nf}\right)+\frac{\partial w_{nf}}{\partial z}=0,$$

(9) $$\begin{array}{l} \frac{1}{\varphi^{2}}\left(v_{nf}\frac{\partial v_{nf}}{\partial r}+w_{nf}\frac{\partial v_{nf}}{\partial z}\right)=-\frac{1}{\rho_{nf}}\frac{\partial p}{\partial r}-\frac{\nu_{nf}v_{nf}}{K}-\frac{\rho_{nf}C_{f}}{\sqrt{K}}\left|v_{nf}\right|v_{nf}+\\ +\frac{v_{nf}}{\varphi }\left[\frac{1}{r}\frac{\partial }{\partial r}\left(r\frac{\partial v_{nf}}{\partial r}\right)-\frac{v_{nf}}{r^{2}}+\frac{\partial^{2}v_{nf}}{\partial z^{2}}\right], \end{array}$$

(10) $$\begin{array}{l} \frac{1}{\varphi^{2}}\left(v_{nf}\frac{\partial w_{nf}}{\partial r}+w_{nf}\frac{\partial w_{nf}}{\partial z}\right)=-\frac{1}{\rho_{nf}}\frac{\partial p}{\partial z}-\frac{\nu_{nf}w_{nf}}{K}-\frac{\rho_{nf}C_{f}}{\sqrt{K}}\left|v_{nf}\right|w_{nf}+\\ +\frac{v_{nf}}{\varphi }\left[\frac{1}{r}\frac{\partial }{\partial r}\left(r\frac{\partial w_{nf}}{\partial r}\right)+\frac{\partial^{2}w_{nf}}{\partial z^{2}}\right], \end{array}$$

(11) $$\rho_{nf}c_{p,nf}\left(v_{nf}\frac{\partial T_{nf}}{\partial r}+w_{nf}\frac{\partial T_{nf}}{\partial z}\right)=\frac{1}{r}\frac{\partial }{\partial r}\left(rk_{eff}\frac{\partial T_{nf}}{\partial r}\right)+\frac{\partial }{\partial z}\left(k_{eff}\frac{\partial T_{nf}}{\partial z}\right).$$

Here, *K*—wick permeability, $\left|v_{nf}\right|$—magnitude of the liquid velocity, *k_eff_*—effective thermal conductivity, *C_f_*—dimensionless drag constant, φ—porosity of the wick. The following abbreviations are used here nf—nanofluid.

The effective thermal conductivity *k_eff_* captures the integrated contributions of three key factors: the porous geometry of the screen-mesh wick, the thermophysical behavior of the nanofluid, and the interfacial nanolayers enveloping nanoparticles. The *k_eff_* of the nanofluid-saturated wick can be computed using the following relationship:

(12) $$k_{eff}=\frac{k_{nf}\left[k_{nf}+k_{s}-\left(1-\varphi \right)\left(k_{nf}-k_{s}\right)\right]}{\left(k_{nf}+k_{s}\right)+\left(1-\varphi \right)\left(k_{nf}-k_{s}\right)}$$

The following abbreviations are used here *nf*—nanofluid, *s*—solid phase.

Equations (1)–(5) are relevant for laminar steam and heat transfer in the steam channel. Equations (8)–(12) model capillary flow in a porous wick taking into account the effective thermal conductivity of nanofluids.

There are a large number of formulas for calculating the effective thermal conductivity of a nanofluid. Some relationships are based on Maxwell’s approach, some are formulated based on experimental approaches. Let us take as an example a modified Maxwell model [5,6,7]:

(13) $$k_{nf}=k_{bf}\frac{\left[k_{np}+2k_{bf}+2\left(k_{np}-k_{bf}\right){\left(1+\beta \right)}^{3}\phi \right]}{k_{np}+2k_{bf}+2\left(k_{np}-k_{bf}\right){\left(1+\beta \right)}^{3}\phi }$$

(14) $$k_{np}=k_{bp}\frac{\left[2\left(1-\alpha \right)+{\left(1+\beta \right)}^{3}\left(1+2\alpha \right)\right]\alpha }{\left(\alpha -1\right)+{\left(1+\beta \right)}^{3}\left(1+2\alpha \right)}$$

Here, α is the ratio between the thermal conductivity of the deposition layer and the intrinsic thermal conductivity of the nanoparticle material without any deposition, β is the ratio of nanolayer thickness to nanoparticle radius. The following abbreviations are used here *np* is the effective nanoparticle, *bp* is the bulk material of the nanoparticle. It should be noted that the effective thermal conductivity of the nanoparticle can be the same as that of the bulk thermal conductivity.

From another side, it is more suitable to use not theoretical relations for the effective thermal conductivity, but experimentally-based correlations. Thus, Liu et al. [8] have proposed the experimentally-based correlation for the thermal conductivity of carboxymethyl cellulose (CMC) based Fe_3_O_4_ and Al_2_O_3_ nanofluids:

(15) $$k_{nf}=0.00943\phi^{0.43017}{\dot{\gamma }}^{0.8412}+0.0902\phi +0.52732\;(\text{for}\;{\text{Fe}}_{3}O_{4}\;\text{nanoparticles})$$

(16) $$k_{nf}=0.00876\phi^{0.18486}{\dot{\gamma }}^{0.69881}+0.92934\phi +0.50343\;(\text{for}\;{\text{Al}}_{2}O_{3}\;\text{nanoparticles})$$

where ϕ is a nanoparticles volume fraction and $\dot{\gamma }$ is shear rate.

Selvarajoo et al. [9] have studied experimentally the thermal conductivity of Al_2_O_3_-GO mixed with host fluid (deionized water) at 0.25%, 0.5%, 0.75% and 1.0% of volume concentration (ϕ) and temperature (*T*) between 30 °C and 50 °C and the following relation has been received

(17) $$k_{nf}=0.138\phi +0.003T+0.66$$

Ghafouri and Toghraie [10] have scrutinized experimentally the SiC-MgO/EG (50–50%) hybrid nanofluid thermal conductivity and extracted the following relation

(18) $$\frac{k_{nf}}{k_{f}}=1.01859{\left(1+0.01\phi \right)}^{9.785}{\left(1+0.02T_{nf}\right)}^{0.077}{\left(1+d_{np}/90\right)}^{-0.059}$$

where ϕ is the nanoparticles volume fraction up to 1%, *T_nf_* is the temperature of hybrid nanofluid between 25 °C and 50 °C, and *d_np_* is the diameter of the nanoparticles between 20 nm and 90 nm.

Ajeena et al. [11] have studied experimentally the thermal conductivities of two nanofluids, namely, distilled water (DW) with nanoparticles of zirconium oxide (ZrO_2_) and DW with silicon carbide (SiC) nanoparticles for temperatures (*T*) between 20 °C and 60 °C and nanoparticles volume fraction (ϕ) up to 0.1%. The following relations have been extracted

(19) $$\frac{k_{nf}}{k_{bf}}=0.00146\cdot T+1.60971\cdot \phi +0.9584\;(\text{for}\;{\text{ZrO}}_{2}/\text{DW})$$

(20) $$\frac{k_{nf}}{k_{bf}}=0.00256\cdot T+2.14143\cdot \phi +0.96749\;(\text{for}\;\text{SiC}/\text{DW})$$

Relationships for the density, viscosity and specific heat capacity of a nanofluid can be obtained from various types of correlations obtained empirically or experimentally. Below there are some variants of these relationships [5,12,13]:

(21) $$\begin{array}{l} \rho_{nf}=\left(1-\phi \right)\rho_{bf}+\phi \rho_{np},\\ \mu_{nf}=\frac{\mu_{bf}}{{\left(1-\phi \right)}^{2.5}},\\ C_{p,nf}=\frac{\left(1-\phi \right)+\left(\rho_{bf}C_{p,bf}\right)+\phi \left(\rho_{np}C_{p,np}\right)}{\left(1-\phi \right)\rho_{bf}+\phi \rho_{np}}. \end{array}$$

where $\phi =V_{np}/\left(V_{bf}+V_{np}\right)$ is the nanoparticles volume fraction.

Thus, Selvarajoo et al. [9] have studied experimentally the dynamic viscosity of Al_2_O_3_-GO mixed with deionized water at 0.25%, 0.5%, 0.75% and 1.0% of volume concentration (ϕ) and temperature (*T*) between 30 °C and 50 °C and the following relation has been received

(22) $$\mu_{nf}=0.275\phi -0.0027T+0.887$$

Ajeena et al. [14] have investigated experimentally the mono nanofluids dynamic viscosity of distilled water filled with nanoparticles of zirconium oxide and silicon carbide for temperature (*T*) between 20 °C and 60 °C and nanoparticles volume fraction (ϕ) up to 0.1%. The following relations have been extracted

(23) $$\begin{array}{l} \frac{\mu_{nf}}{\mu_{bf}}=0.99761+0.26995\cdot \phi^{0.32737}-0.03587\cdot T^{0.89391}+\\ +0.19267\cdot \phi^{0.32737}\cdot T^{0.89391} \end{array}\;(\text{for}\;{\text{ZrO}}_{2}/\text{DW})$$

(24) $$\begin{array}{l} \frac{\mu_{nf}}{\mu_{bf}}=62.5681-16202.79566\cdot \phi^{1.23096}-61.32394\cdot T^{0.00072885}+\\ +16172.3693\cdot \phi^{1.23096}\cdot T^{0.00072885} \end{array}\;(\text{for}\;\text{SiC}/\text{DW})$$

Bhat and Qayoum [15] have received the experimentally-based relation for the dynamic viscosity of ethylene glycol with CuO nanoparticles

(25) $$\frac{\mu_{nf}}{\mu_{b}}=3.5162\cdot \phi^{0.2636}{\left(T_{nf}/T_{b}\right)}^{-9.2}{\left(d_{p}/d_{b}\right)}^{0.0018}$$

where *T_b_* is the reference temperature (*T_b_* = 293.2 K), *d_p_* is size of the nanoparticles between 15 nm and 75 nm, *d_b_* is the size of the base fluid molecule, ϕ is the nanoparticles volume fraction between 1% and 4%.

Other interesting correlations can be found in published review papers [16,17].

In recent years, a significant number of studies have emerged on the use of artificial intelligence to predict the viscosity of nanofluids [18,19,20,21]. These investigations highlight the growing relevance of AI-driven approaches in enhancing the accuracy and efficiency of such predictions. This topic is highly actual due to its potential applications in optimizing industrial processes and advancing material science.

Finally, functional dependencies for nanofluids in heat pipes are abundant and context-dependent, shaped by the interplay of physics at molecular, colloidal, and macroscopic scales. Researchers must judiciously select models aligned with their specific nanofluid system, prioritizing validated correlations for targeted parameters. Future work should focus on unifying these fragmented dependencies into standardized frameworks, possibly leveraging machine learning to handle multi-variable interactions.

Despite significant progress in modeling of heat pipes with nanofluids, key challenges and unresolved issues remain that require further research. For example, multiphase interactions in nanofluids require detailed elaboration. Existing models (e.g., modified Maxwell’s equations for thermal conductivity) do not always account for nanoparticle aggregation, changes in surface wettability, or the formation of nanolayers during evaporation/condensation. One possible solution is to develop hybrid models that combine molecular dynamics for nanoscale effects and continuum approaches for the macroscale.

Another challenge is the heterogeneity of the wick porous structures. The Darcy-Brinkman equations assume uniform porosity, but real wicks (e.g., composite or sintered) have a hierarchical structure with varying permeability. One possible solution is to use digital twin techniques to create 3D models of wicks based on micro-CT scanning.

The long-term stability of nanofluids is also a topical issue. Experimental correlations for viscosity (21)–(25) are valid for short experiments, but particles sedimentation and degradation of properties during long-term operation are poorly understood. Long-term tests with in-situ monitoring (e.g., spectroscopy methods) and modeling of nanofluid aging are recommended for future studies.

This section provides a comprehensive overview of the mathematical models governing fluid flow, heat transfer, and phase-change phenomena in heat pipes, with a focus on nanofluid-enhanced systems. The governing equations for vapor flow, wick transport, and heat conduction are presented, emphasizing continuum and porous media approaches.

## 3. Peculiarities in Description of Transport Phenomena in Wicks

Wick-based heat pipes utilize capillary action to circulate working fluid. Wick (usually porous structure) returns condensate from the condenser to the evaporator, overcoming gravity and flow resistance. Modeling of transport processes in wicks and the choice of approaches to their description raise some questions among researchers. There is no unified data on the design and properties of the wick in the available literature.

Describing transporting processes in wicks involves some challenges due to their complex microstructure, multiphase interactions, and coupled phenomena.

### 3.1. Porous (Composite) Wick

**Figure 2:**
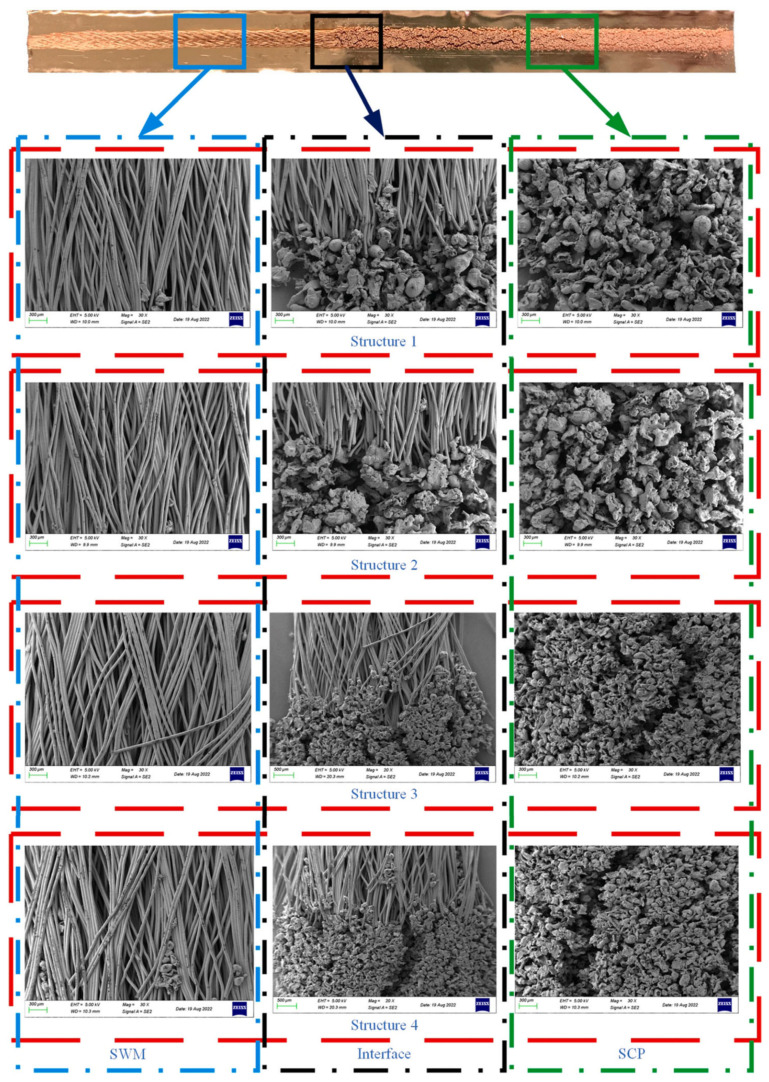
An example of the calculation domain of a heat pipe with a composite (copper) wick. Reproduced from ([22]). Copyright ©2025 Elsevier Masson SAS. All rights reserved.

**Figure 3:**
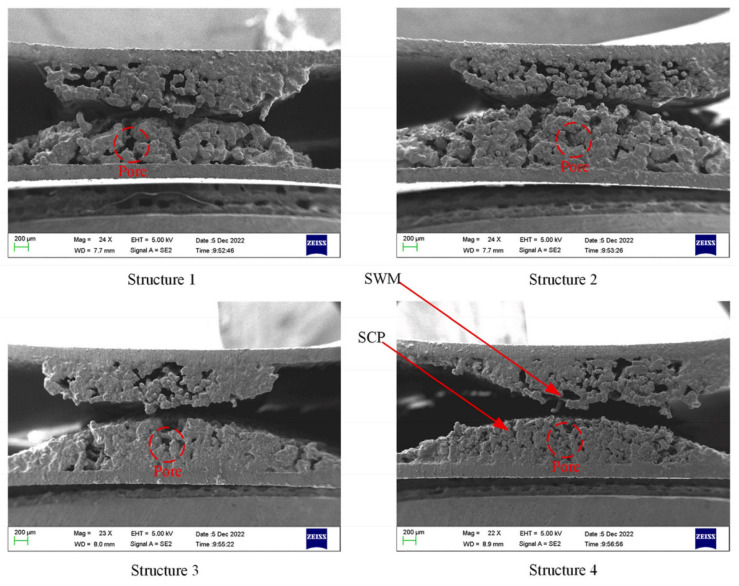
Double-layer as wick in heat pipe. Reproduced from ([23]). Copyright ©2025 Elsevier Masson SAS. All rights reserved.

A porous (composite) wick is a structured material designed to enhance capillary-driven fluid transport in thermal management systems, such as heat pipes or vapor chambers. It combines multiple materials or hierarchical pore structures (e.g., sintered metals, carbon fibers, or polymer matrices) to balance permeability and capillarity, optimizing liquid replenishment and vapor escape. Composite wicks often integrate coarse pores to reduce flow resistance and fine pores to maximize capillary pressure, enabling efficient phase-change heat transfer. Their tailored geometry and surface modifications improve wettability, thermal conductivity, and resistance to dry-out under high heat fluxes. For example, Yi et al. [22] have considered hydrodynamic behavior in segmented composite for ultra-thin heat pipes, analyzing capillary pressure gradients, permeability trade-offs, and localized flow resistance at porous media interfaces under varying length ratios and particle sizes (see Figure 2). Optimal hydrodynamic performance was observed at a 50% SCP-SWM ratio, where synergistic capillary action (SWM) and permeability (SCP) minimized pressure losses and enhanced liquid-vapor circulation, despite challenges in balancing pore-scale flow uniformity. Segmented wicks achieved a 16.7% higher heat dissipation capacity than single-layer structures, driven by reduced interfacial flow disruptions and improved dynamic stability of the working fluid across heterogeneous porous domains. Yi et al. [23] have experimentally investigated the hydrodynamic interplay in a novel double-layer composite wick (sintered copper powder and spiral woven mesh) for ultra-thin heat pipes, revealing that optimized particle size gradients in the sintered layer enhance capillary pressure while maintaining permeability, thereby reducing interfacial flow resistance and promoting efficient liquid-vapor circulation (see Figure 3). The composite wick demonstrated a 16.7% increase in maximum heat dissipation capacity compared to single-layer structures, attributed to synergistic hydrodynamic interactions between the high-permeability copper powder and the high-capillary-action woven mesh, which minimized localized pressure drops at porous media junctions. Directional heating tests further highlighted asymmetric hydrodynamic responses, with heat applied to the sintered layer yielding lower thermal resistance (0.069 °C/W) due to enhanced nucleation sites and vapor-liquid phase-change dynamics, underscoring the critical role of pore-scale flow uniformity in composite wick performance. Wang et al. [24] have studied the hydrodynamic optimization of ultra-thin flat heat pipes (UTFHPs) with multiscale striped composite wicks, demonstrating that a 1 mm vapor-liquid channel width in copper foam paired with superhydrophilic mesh layers minimizes interfacial flow resistance by segregating vapor and liquid pathways, thereby enhancing capillary-driven reflux and reducing thermal resistance to 0.79 °C/W at 23.34 W. The striped configuration directs liquid working fluid convergence toward the heating zone while expanding vapor dispersion channels, mitigating counterflow interactions and pressure drops, which is critical for managing localized heat fluxes in compact electronics. Hydrophilic nanostructured surfaces on the composite wick further amplify capillary forces, enabling rapid liquid replenishment and stable two-phase circulation, underscoring the interplay between pore-scale geometry and macroscale hydrodynamic efficiency in thermal management.

Xiong et al. [25] have employed a novel composite wick structure for loop heat pipes (LHPs), combining sintered copper and ceramic powders through low-temperature pressureless sintering, to mitigate heat leakage into the compensation chamber and enhance hydrodynamic performance by optimizing capillary-driven fluid circulation. The composite wick leverages the high thermal conductivity of copper for efficient evaporation and the ultra-low thermal conductivity of ceramic to suppress parasitic heat transfer, while its dual-layer architecture balances capillary pressure and flow resistance to sustain stable working fluid replenishment.

Experimental results demonstrate that the composite wick LHP achieves successful startup at ≥30 W, with maximum heat loads of 550 W (water-cooled) and 500 W (air-cooled), exhibiting lower junction temperatures and thermal resistances compared to mono-wick LHPs under moderate power inputs, validating its superior hydrodynamic and thermal management capabilities.

Ming et al. [26] have investigated the hydrodynamic performance of ultra-thin flat heat pipes (UFHPs) with composite wicks comprising copper fiber bundles and mesh layers, emphasizing the interplay between capillary-driven liquid flow and vapor pressure dynamics. The study revealed that heterogeneous wick structures, combining variable fiber diameters and single-layer meshes, enhance capillary pressure by optimizing porosity and permeability, thereby reducing hydraulic resistance and improving liquid return efficiency. Experimental results demonstrated a 6 W increase in maximum heat transfer capacity compared to homogeneous designs, attributed to stabilize two-phase flow and reduced viscous shear stress in vapor channels under high thermal loads.

Zhang et al. [27] have developed a three-dimensional transient numerical model of a flat micro heat pipe (FMHP) with a composite wick of copper foam and spiral woven mesh. The model integrated the Volume of Fluid (VOF) method and Leverett function to simulate capillary-driven liquid-vapor interactions in unsaturated porous media. The heterogeneous porosity of the composite wick improved permeability by 23% and reduced thermal resistance by 16% at a 30° inclination, effectively mitigating dry-out effects. Experimental validation confirmed the model’s accuracy at heat fluxes up to 71,111 W/m^2^, highlighting its relevance for thermal management in compact electronic systems.

Ma et al. [28] have explored the hydrodynamic and thermal performance of a loop heat pipe (LHP) employing a carbon sphere-modified nickel biporous wick (CSs-Ni-Wick). The macropores in the structure minimized flow resistance, while micropores enhanced capillary-driven liquid replenishment. Surface hydrophilization (contact angle: 25.8° vs. 67.5° for unmodified wicks) increased vapor nucleation site density, stabilizing the vapor-liquid interface dynamics, particularly under low heat loads. Experiments achieved a maximum heat flux of 20 W/cm^2^ with a minimum thermal resistance of 0.357 °C/W, attributed to suppressed flow stagnation and improved wick wettability.

### 3.2. Grooved Wick

A grooved wick is a capillary structure featuring engineered channels or grooves, typically machined or etched into a substrate, to facilitate liquid transport via capillary action in thermal systems like heat pipes. These grooves act as preferential pathways for working fluid movement, balancing low hydraulic resistance with sufficient capillary pressure to sustain fluid circulation during phase-change heat transfer. Common designs include axial, radial, or spiral grooves, often optimized for specific orientations or gravitational conditions to enhance liquid return from the condenser to the evaporator. Compared to sintered or composite wicks, grooved structures offer simpler fabrication, reduced flow resistance, and improved thermal contact with heated surfaces. However, their performance may depend on groove geometry (depth, width, spacing) and working fluid wettability, limiting effectiveness in high heat flux or adverse gravity scenarios without additional modifications.

**Figure 4:**
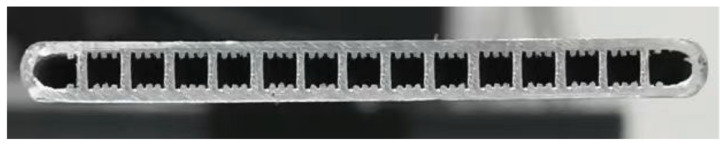
Heat pipe with grooved wick (experiment). Reproduced from ([29]). Copyright ©2025 Elsevier Masson SAS. All rights reserved.

For example, Liu et al. [29] have presented the results of the hydrodynamic behavior of liquid flow within grooved wicks, emphasizing capillary-driven transport mechanisms and phase-change interactions (see Figure 4). The analysis highlights the influence of groove geometry, surface wettability, and fluid properties on the stability and efficiency of liquid distribution. The findings provide insights into optimizing wick structures for enhanced thermal performance in heat pipes and other passive cooling applications.

**Figure 5:**
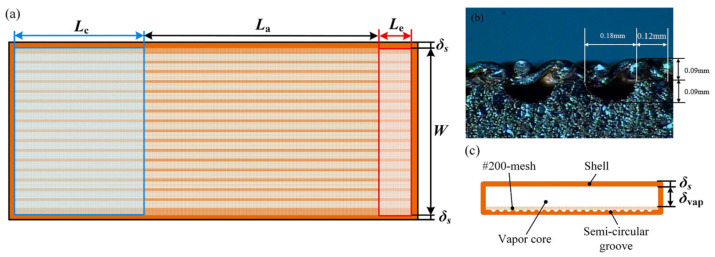
Heat pipe with grooved wick (numerical simulation): (**a**) overall schematic; (**b**) the composite mesh-groove wick; (**c**) side view. Reproduced from ([30]). Open Access Article.

Xiong et al. [30] have developed the hydrodynamic performance of ultra-thin heat pipes (UTHPs) with hybrid mesh-grooved wicks, focusing on the interplay between groove permeability and capillary-driven fluid dynamics, where grooved wicks reduce liquid flow resistance while mesh enhances capillarity. Numerical simulations reveal that partial mesh coverage over the evaporator section optimizes vapor-liquid circulation by expanding vapor space, lowering vapor velocity (up to 25.1% reduction at φ = 122%), and minimizing pressure drops, thereby enhancing mass flow efficiency and thermal uniformity. The validated theoretical model demonstrates that hybrid wick designs, particularly evaporator-focused mesh coverage, reduce thermal resistance by 48.9% under constrained vapor core conditions, highlighting the critical role of hydrodynamic balance in optimizing phase-change heat transfer for high-power electronics (see Figure 5).

Results of study of the hydrodynamic characteristics of liquid flow in grooved wicks, focusing on capillary-driven transport and the interplay between surface tension and viscous forces have been shown by Wang et al. [31]. The analysis highlights the effects of groove geometry, liquid properties, and wetting conditions on flow stability and efficiency. The findings contribute to the optimization of wick structures for enhanced performance in heat transfer applications, particularly in heat pipes and passive cooling systems.

Wang et al. [32] have investigated the hydrodynamic and thermal performance of flat-plate micro heat pipes (FPMHPs) incorporating composite wick structures composed of micro-fin grooves and copper foam, focusing on capillary-driven fluid dynamics under varying gravity conditions. The integration of micro-fin grooves enhances capillary forces by optimizing liquid-vapor interfacial dynamics, while copper foam with tailored pore diameters (0.8 mm) and porosity (80%) balances permeability and capillary pressure, enabling efficient condensate return even in anti-gravity orientations. Experimental results demonstrate that the composite wick structure achieves up to 4.57 higher effective thermal conductivity than non-foam designs, with optimal performance at inclination angles between 0° and −10°, highlighting its suitability for aerospace thermal management under dynamic hydrodynamic constraints.

Wang et al. [33] have examined the hydrodynamic performance of micro heat pipe arrays (MHPAs) with composite wicks integrating copper foam and rectangular micro-fins, focusing on capillary-driven liquid reflux and vapor flow dynamics under varying pore diameters (110–130 PPI) and porosities (67–85%). Experimental results demonstrate that larger pore diameters and higher porosity in copper foam reduce vapor flow resistance and enhance condensate return via improved capillary action, achieving up to 38% lower thermal resistance compared to conventional MHPAs. The interplay between micro-fin geometry and foam structure optimizes phase-change equilibrium, balancing evaporation-conduction rates while mitigating hydrodynamic bottlenecks such as vapor accumulation and condensate reflux delays under diverse ambient temperatures.

### 3.3. Screen Mesh Wick

A screen mesh wick is a type of capillary structure used in heat pipes and vapor chambers for efficient thermal management. It consists of fine metal mesh layers that facilitate liquid flow through capillary action, ensuring continuous fluid circulation. The high porosity and fine pore size of the mesh enhance heat transfer by promoting rapid liquid redistribution. Common materials for screen mesh wicks include stainless steel, copper, and nickel, selected for their thermal conductivity and durability.

**Figure 6:**
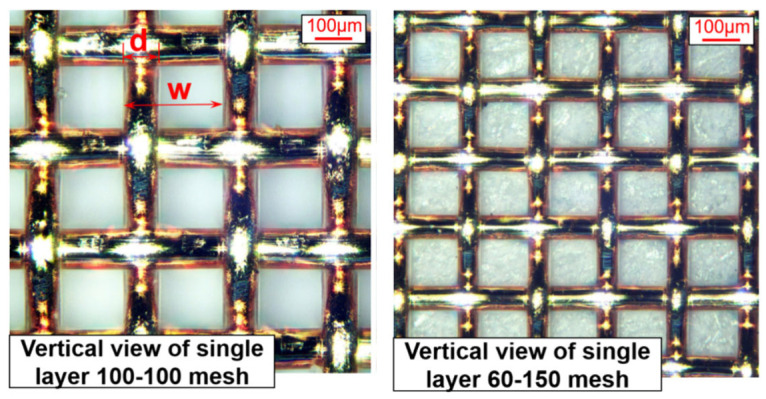
Screen mesh in heat pipe (experiment). Reproduced from ([34]). Copyright ©2025 Elsevier Masson SAS. All rights reserved.

For example, Tan et al. [34] have analyzed the hydrodynamic behavior of liquid flow in screen mesh wicks, emphasizing the capillary-driven transport and interactions between permeability and surface tension (see Figure 6). The research highlights the effects of mesh porosity, fiber diameter, and liquid properties on flow resistance and distribution uniformity. The findings contribute to optimizing wick structures for improved thermal performance in heat pipes and passive cooling systems. Ma et al. [35] have studied the hydrodynamic properties of multilayer screen wicks in heat pipes, focusing on how stacking configurations (A, B, C) influence permeability and flow resistance, with numerical simulations revealing that stable configurations like A optimize capillary-driven fluid dynamics by balancing compactness and reduced hydraulic resistance. Experimental and computational analyses demonstrate that the permeability of screen wicks depends on structural parameters such as wire diameter, mesh number, and layer arrangement, where Configurations A (most stable) dominate flow behavior, minimizing pressure drops while maintaining high capillary forces. The derived universal model, validated across multiple samples, predicts permeability with <10% error by correlating structural ratios (w/d) and statistical distributions of stable configurations, providing critical insights for designing high-efficiency heat pipes with tailored hydrodynamic performance.

**Figure 7:**
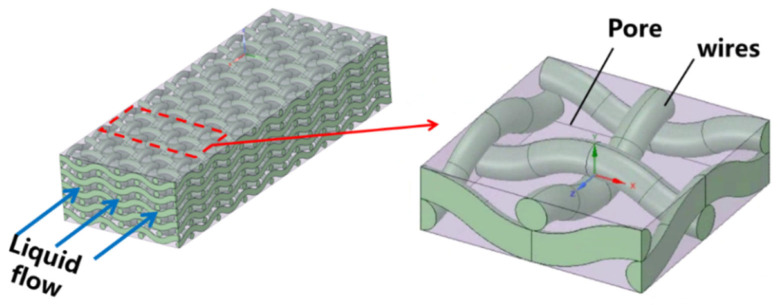
Screen mesh wick in heat pipe (numerical simulation). Reproduced from ([36]). Copyright ©2025 Elsevier Masson SAS. All rights reserved.

Ma et al. [36] have examined a three-dimensional multiscale capillary evaporating film model for screen-wick heat pipes, emphasizing the hydrodynamic interplay between disjoining pressure, surface tension, and wick geometry (see Figure 7). The model divides the liquid film into a capillary pressure microlayer region, governed by molecular forces and curvature, and a macroscopic film shaped by the anisotropic screen-wick structure, revealing that wire spacing and diameter critically influence capillary pressure and flow resistance. Hydrodynamic self-regulation of liquid height balances evaporation and pressure under varying thermal loads, while excessive heat flux disrupts this equilibrium, triggering capillary pressure decline and localized drying due to mismatched flow resistance and interfacial dynamics.

Ma et al. [37] have presented data of the two-dimensional heat and mass transfer model for sodium screen-wick heat pipes, emphasizing hydrodynamic interactions between capillary forces, liquid-vapor flow resistance, and wick geometry under varying inclinations and heat fluxes. The model integrates compressible vapor dynamics, incompressible Darcy-Brinkman liquid flow in the anisotropic screen wick, and iterative gradient descent methods to resolve three-dimensional meniscus curvature, revealing that reduced wire spacing amplifies viscous resistance, lowering the capillary limit by 90% when spacing decreases from 80 μm to 15 μm. Experimental validation highlights gravity-driven hysteresis and drying oscillations in inclined pipes, where positive angles induce periodic rewetting due to competing capillary and gravitational forces, while hydrodynamic instabilities in the wick microstructure govern temperature fluctuations and critical heat flux degradation. Tian et al. [38] have studied experimentally the hydrodynamic behavior of liquid sodium in stainless steel and molybdenum screen wicks, revealing that capillary dynamics are governed by irreversible oxide layer destruction at ~460 °C, which enhances wettability and alters mass-height correlations during thermal cycling. The hydrodynamic performance, driven by surface tension and density variations, is predominantly influenced by mesh size and interlayer spacing, with molybdenum wicks exhibiting superior capillary rise (up to 18.2 cm) due to reduced chemical reactivity and optimized pore geometry. A validated capillary model demonstrates that hydrodynamic instabilities from microstructural deformations in fine meshes introduce <12% error in predicting sodium mass distribution, emphasizing the critical role of wick anisotropy and thermal history in high-temperature alkali-metal heat pipes.

**Figure 8:**
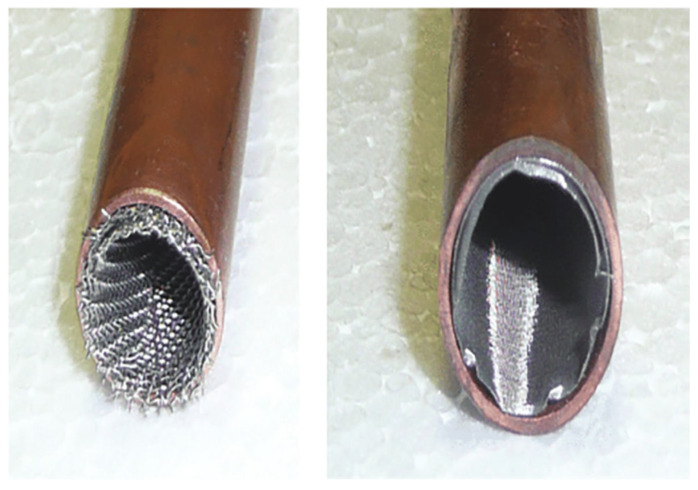
Screen mesh wick in heat pipe (experiment). Reproduced from ([39]). Copyright ©2025 Elsevier Masson SAS. All rights reserved.

Nookaraju et al. [39] have presented results of the experimental and numerical study investigates the hydrodynamic performance of a screen mesh wick heat pipe, revealing that capillary-driven flow in dual-layer wicks (mesh sizes 60 and 100) exhibits reduced thermal resistance (0.023–0.042 °C/W) with increasing heat flux due to enhanced molecular mobility and optimized liquid-vapor phase interactions (see Figure 8). Hydrodynamic instabilities, influenced by inclination angles (15–90°) and heat inputs (20–160 W), demonstrate that maximum heat transfer coefficients (8800 W/m^2^·K in ANSYS vs. 8500 W/m^2^·K experimentally) occur at 60 W, attributed to minimized viscous losses and efficient capillary pumping in the anisotropic wick structure. Numerical simulations predict lower thermal gradients compared to experiments, highlighting the role of convective heat losses and wick-fluid interfacial dynamics in real-world hydrodynamic performance.

Liu et al. [40] have studied the hydrodynamic behavior in ultrathin heat pipes with copper mesh wicks, focusing on the interplay between filling rate, capillary pressure, and liquid-vapor phase dynamics in confined geometries. A steady-state distributed parameter model reveals that unsaturated wick conditions enable self-regulating capillary pressure adjustments, balancing liquid film thickness and vapor flow resistance, with optimal filling rates (e.g., 0.6) minimizing thermal resistance by mitigating evaporator dry-out and condenser subcooling. The analysis highlights how mesh specifications and interfacial shear stresses influence hydrodynamic performance, with finer meshes enhancing capillary-driven liquid redistribution but increasing vapor flow friction, ultimately dictating heat transfer limits under varying operational fluxes.

### 3.4. Sintered Wick

A sintered wick is a porous capillary structure used in heat pipes and vapor chambers to facilitate efficient liquid transport and heat transfer. It is manufactured by sintering metal powders, typically copper or stainless steel, to create a highly porous network with controlled pore size and permeability. The fine interconnected pores enhance capillary pumping, allowing for effective liquid redistribution even against gravity. Due to its high capillary pressure and thermal conductivity, the sintered wick is widely utilized in high-performance thermal management applications, including electronics cooling and aerospace systems. Compared to other wick structures, it offers superior heat transfer efficiency but may have higher manufacturing complexity and cost.

**Figure 9:**
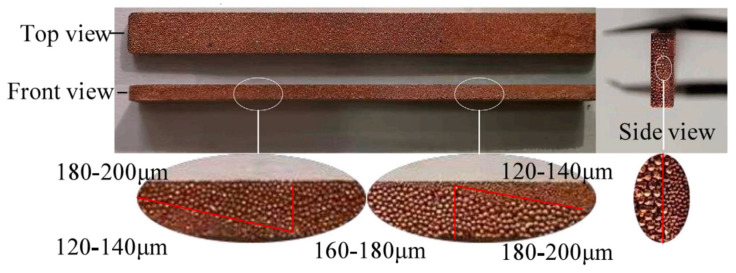
Sintered wick in heat pipe (experiment). Reproduced from ([41]). Open Access Article.

For example, the hydrodynamic and thermal performance of a flat micro-heat pipe (FMHP) employing a sintered multi-size copper powder wick (SMW), featuring a bilayer structure in the evaporator and condenser sections to enhance capillary pressure and permeability (see Figure 9) have been analyzed by Zhao et al. [41]. The SMW’s graded pore design optimizes vapor-liquid dynamics by promoting rapid bubble escape in the evaporation layer (via larger upper pores) and efficient condensation (via smaller upper pores in the condenser), while the adiabatic section maintains balanced fluid transport. Experimental results demonstrate a 16.59% reduction in liquid transfer time and a 14.22% lower thermal resistance compared to single-size wicks, attributed to improved hydrodynamic interactions, including enhanced capillary-driven reflux and reduced flow resistance in the multi-scale porous structure.

Wang and Wan [42] have investigated the hydrodynamic and thermal performance of a stainless-steel heat pipe with a sintered stainless-steel fiber wick, emphasizing the role of fiber microstructure (turned vs. drawn fibers) and working fluid wettability on capillary-driven fluid dynamics. The turned fiber wick, featuring micro-scale surface structures, enhances capillary pressure and permeability, particularly when paired with water-ethanol solutions (optimal concentration ratios of 9:1 to 13:1), reducing flow resistance and improving anti-gravity heat transfer. Experimental results demonstrate a 14.22% reduction in thermal resistance and increased power limits under adverse inclination, attributed to improved wettability and synergistic interplay between pore structure dynamics and ethanol-water interfacial properties.

Maneemuang et al. [43] have experimentally performed the hydrodynamic effects of pipe flattening on vapor core pressure drop and thermal performance in miniature heat pipes with sintered fiber wicks, focusing on how reduced vapor core dimensions and altered flow dynamics impact heat transfer. Experimental and numerical analyses reveal that flattening increases vapor velocity and pressure drop due to restricted vapor core cross-sectional area, with critical thickness thresholds (e.g., 0.45 mm for a 2 mm pipe) leading to significant thermal resistance spikes attributed to diminished vapor flow efficiency and wick deformation. The proposed normalized parameter identifies a critical flattening limit (0.06) to optimize vapor core retention, offering design guidelines to mitigate hydrodynamic losses while maintaining thermal performance in compact electronic cooling applications.

Sangpab et al. [44] have analyzed the hydrodynamic impacts of bending and flattening on cryogenic sintered-wick heat pipes, revealing that deformation of the sintered porous structure disrupts capillary-driven liquid return and increases hydraulic resistance, particularly under bending angles up to 90° and reduced final thicknesses. Experimental results demonstrate that flattening to critical thicknesses (e.g., 2.5 mm) collapses the wick’s pore network, causing liquid clogging in the condenser and reducing vapor core area, which elevates thermal resistance by up to 155% compared to cylindrical counterparts. The combined bending-flattening effects exacerbate hydrodynamic inefficiencies, with sintered wick deformation and restricted vapor-liquid interfacial dynamics identified as primary contributors to degraded heat transfer performance in compact cryogenic cooling applications.

**Figure 10:**
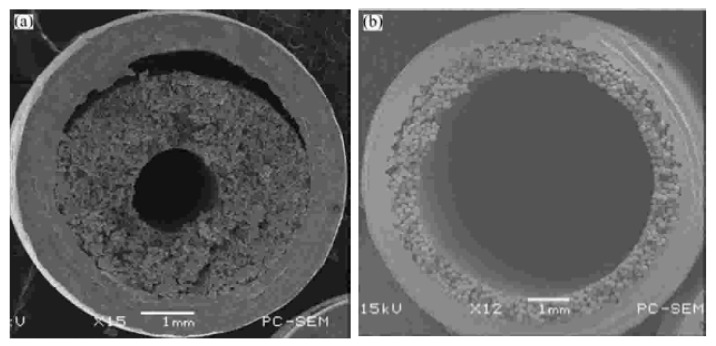
Sintered wick after experiment ((**a**) horizontal sintering, (**b**) vertical sintering). Reproduced from ([45]). Copyright ©2025 Elsevier Masson SAS. All rights reserved.

The hydrodynamic characteristics of sintered wicks in miniature cylindrical heat pipes, focusing on optimizing sintering parameters (temperature, time, atmosphere, and orientation) to enhance capillary-driven fluid transport have been examined (see Figure 10) by Jiang et al. [45]. Key hydrodynamic features include porosity-dependent capillary limits, permeability influenced by radial shrinkage, and microcracks formed during redox reactions, which augment surface area and liquid circulation efficiency. The optimized sintering process (950 °C for 159 µm powders, 3 h for 0.45–0.6 mm wicks) balances porosity (47.7%) and radial shrinkage (3.68%), minimizing thermal resistance while maintaining effective capillary pumping and anti-gravity performance.

Ginting et al. [46] have studied the hydrodynamic performance of sintered stainless steel metal foam wicks in cylindrical heat pipes, focusing on surface modifications (superhydrophilic & superhydrophobic) and their impact on capillary-driven fluid dynamics. Experimental results reveal that superhydrophilic-treated metal foams exhibit enhanced capillary pressure (0.65 Pa) and rapid liquid absorption due to reduced pore resistance (average pore size: 443 µm, 30 PPI), optimizing condensate reflux and vapor-liquid phase equilibrium. The optimized wick structure achieved a thermal resistance of 2.47 °C/W at 5 W, demonstrating improved heat transfer efficiency through tailored pore geometry and wettability-driven hydrodynamic interactions.

Sun et al. [47] have investigated the hydrodynamic performance of micro-fin sintered wicks in heat pipes, focusing on stainless steel fiber mat and nickel foam wicks modified via oxidation-reduction to enhance nanostructured surfaces. The results demonstrate that nanostructured wicks exhibit superior hydrophilicity, increasing the capillary performance factor (M) by up to 59.1% due to reduced effective capillary radius (14.9%) and enhanced permeability (34.7%), which collectively mitigate flow resistance and improve liquid replenishment dynamics. Increased porosity introduces a trade-off, where permeability gains are offset by a dominant rise in effective capillary radius, reducing M by 17.1%, while optimized nanostructuring lowers thermal resistance by 38.1%, underscoring the critical role of surface microstructure in governing hydrodynamic and thermal efficiency in heat pipe wicks.

Guo et al. [48] have examined the hydrodynamic behavior of self-rewetting fluids (SRWF) in gravity heat pipes (GHPs) with sintered porous wicks, revealing that the optimal filling ratio (23%) balances capillary-driven liquid distribution and minimizes dry-out regions by enhancing fluid rewetting through the Marangoni effect. The SRWF, exhibiting a non-linear surface tension-temperature relationship, promotes spontaneous fluid migration toward high-temperature zones under thermal gradients, significantly improving evaporator heat transfer coefficients (53.6% increase at 660 W) and temperature uniformity (66% enhancement at 500 W) compared to water. Infrared visualization confirmed that SRWF mitigates hydrodynamic instabilities in sintered wicks by sustaining thin-film evaporation and nucleate boiling, even under elevated thermal loads, due to synergistic Marangoni and capillary forces.

Yang et al. [49] have experimentally demonstrated that surface-treated metal foam wicks with dense microstructures enhance hydrodynamic performance in flat plate heat pipes (FPHPs) by optimizing capillary-driven liquid redistribution and vapor-liquid phase change dynamics, particularly under anti-gravity conditions. The oxidation-reduction treatment creates microscale surface roughness, which strengthens capillary forces and reduces vapor flow resistance, enabling a 30–50% reduction in thermal resistance and up to 373-fold improvement in effective thermal conductivity compared to copper across varying inclinations. Infrared visualization and thermocouple data confirm that the microstructured wick mitigates dry-out by sustaining uniform fluid distribution and rapid vapor propagation, critical for high-power thermal management in compact electronic systems.

### 3.5. Other Types of Wicks

In addition, there are other methods for describing and modeling the wick. For example, a layer of nanoparticles can be used as a wick. A nanoparticle layer wick is an engineered capillary structure formed by depositing nanoparticles (e.g., silica, alumina) onto the inner surface of a heat pipe or vapor chamber. This ultrathin wick (typically ~10 μm thick) leverages the unique properties of nanoparticles to enhance fluid transport and heat transfer efficiency. For example, Brusly et al. [50] have experimentally examined the hydrodynamic and thermal performance of a heat pipe with a nanoparticle-coated sintered wick, focusing on enhanced capillary-driven fluid dynamics through increased surface porosity and nucleation sites. The nanoparticle coating (80–90 nm Cu) reduces evaporator thermal resistance by 40% due to smaller bubble nucleation and improved boiling efficiency, while increased surface roughness elevates condenser resistance by altering liquid-vapor phase interaction dynamics. Despite higher condenser resistance, the net thermal resistance decreases by up to 19% under 100 W, demonstrating the hydrodynamic trade-offs between enhanced evaporation and compromised condensation in modified wick structures.

**Figure 11:**
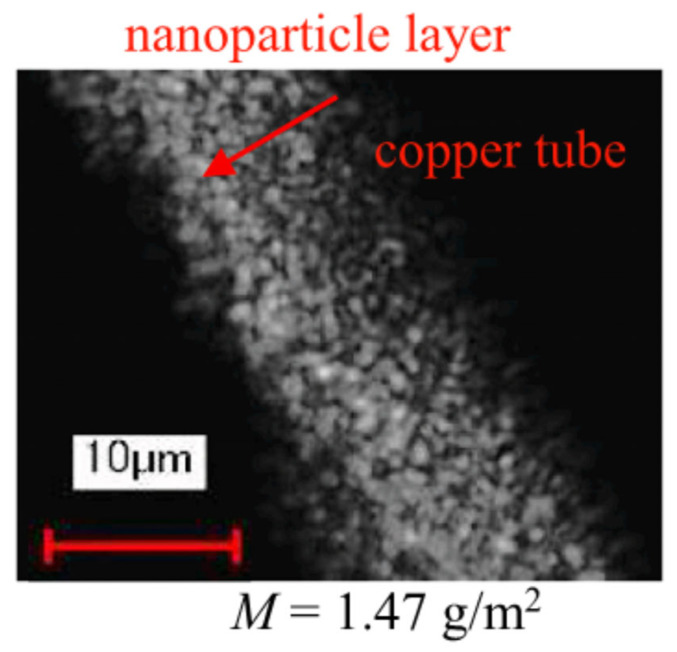
Extended microscope image of the nanoparticle layer formed on the copper tube inner wall for a wicked heat pipe. Reproduced from ([51]). Copyright ©2025 Elsevier Masson SAS. All rights reserved.

Wang et al. [51] have explored the hydrodynamic and thermal performance of heat pipes utilizing a 10 mm-thick silica nanoparticle layer as an ultrathin wick, demonstrating its capillary-driven fluid redistribution and enhanced thermal efficiency compared to traditional mesh wicks (see Figure 11). The nanoparticle layer’s sparse, porous structure (porosity > 0.9) facilitated effective liquid transport, reducing thermal resistance by 63% (0.62 to 0.23 K/W) while maintaining a competitive maximum heat transfer rate (78–120 W), attributed to improved capillarity and uniform surface coverage. Hydrodynamic stability was observed even under repeated dry-out conditions, with nanofluid suspensions aiding nanoparticle layer repair, highlighting the interplay between capillary dynamics, nanoparticle deposition uniformity, and phase-change efficiency in miniaturized thermal management systems.

### 3.6. Comparative Analysis: Mathematical Models & Experimental Results

1. Composite wicks:
  - Models: Continuum approaches (e.g., Darcy-Brinkman equations) and Volume of Fluid (VOF) methods simulate capillary-driven flow in porous media. Zhang et al. [27] developed a 3D transient model predicting 23% permeability improvement, validated experimentally up to 71,111 W/m^2^.
  - Experiments: Yi et al. [23] observed a 16.7% heat dissipation increase in double-layer wicks, aligning with model predictions of reduced interfacial resistance. Discrepancies arise in pore-scale uniformity, often oversimplified in simulations.
2. Grooved wicks:
  - Models: Numerical simulations (Xiong et al. [30]) optimize groove-mesh configurations, predicting 25.1% vapor velocity reduction.
  - Experiments: Hybrid wicks achieved 48.9% lower thermal resistance, validating model insights. However, geometric sensitivity (e.g., groove depth) is less captured in theoretical frameworks.
3. Screen mesh wicks:
  - Models: Multiscale capillary models (Ma et al. [28]) integrate disjoining pressure and surface tension, predicting hydrodynamic instabilities with <12% error.
  - Experiments: Nookaraju et al. [39] reported thermal resistance reduction (0.023–0.042 °C/W) under increasing heat flux, consistent with model trends. However, numerical simulations often underestimate convective losses.
4. Sintered wicks:
  - Models: Pore-network models predict capillary limits and permeability trade-offs. Jiang et al. [45] optimized sintering parameters (porosity: 47.7%) to minimize thermal resistance.
  - Experiments: Surface-treated wicks (Ginting et al. [46]) achieved 0.65 Pa capillary pressure experimentally, aligning with predictions. Flattening-induced pore collapse (Sangpab et al. [44]) caused 155% thermal resistance spikes, highlighting model limitations in deformation effects.
5. Nanoparticle-enhanced wicks:
  - Models: Thin-film evaporation models (Brusly et al. [50]) predict 40% evaporator resistance reduction but struggle with nanoparticle agglomeration dynamics.
  - Experiments: Wang et al. [51] confirmed 63% resistance reduction but noted coating instability under prolonged operation, underscoring gaps in long-term modeling.

Key Findings:

- Agreements: Most models accurately predict trends in capillary pressure, permeability, and thermal resistance reduction. Experimental validations confirm the efficacy of hybrid/composite designs and surface modifications.
- Discrepancies: Simplifications in pore-scale interactions, nanoparticle stability, and deformation effects (e.g., bending/flattening) lead to deviations. Experimental setups often face uncontrollable variables (e.g., wettability hysteresis) not fully captured in simulations.
- Recommendations: Unified frameworks integrating multiscale modeling (microstructure to macroscale) and long-term stability assessments are needed to bridge theory-practice gaps.

In addition, Table 1 and Table 2 show a comparison of the characteristics of the heat pipe processes for different types of wicks and based fluid.

These Tables synthesize experimental and theoretical insights, providing a practical roadmap for selecting wicks and nanofluids based on application-specific requirements. Based on the information provided, it can be generalized following way:

1. Composite wicks excel in high heat flux scenarios but require advanced manufacturing.
2. Grooved wicks are cost-effective for low-power applications but need geometry optimization.
3. Nanoparticle-enhanced wicks show exceptional thermal gains but demand stabilization techniques (e.g., surface functionalization).
4. Sintered wicks are durable but face scalability challenges; deformation-aware models are critical.
5. Self-rewetting nanofluids (e.g., SRWF) demonstrate superior thermal management under gradients but require long-term stability tests.
6. Future work:
  - Standardize testing protocols for wick-nanofluid combinations.
  - Develop AI-driven models to predict optimal configurations.
  - Prioritize green nanofluids (e.g., cellulose-based) for sustainable applications.

### 3.7. Current Challenges and Future Research Directions

Section 3 critically examines the hydrodynamic and thermal performance of various wick structures in heat pipes, emphasizing their design complexities, capillary-driven fluid dynamics, and phase-change interactions. Key wick types include:

- Composite (porous) wicks: Engineered with hierarchical pore structures (e.g., sintered metals, copper foam, and mesh layers), these wicks optimize capillary pressure and permeability. Studies (Yi et al. [22,23], Wang et al. [24]) demonstrate enhanced thermal performance (e.g., 16.7% higher heat dissipation) through reduced interfacial flow resistance and improved vapor-liquid circulation.
- Grooved wicks: Simple to fabricate, these wicks rely on channel geometry for low hydraulic resistance but face dry-out risks under high heat fluxes. Hybrid designs (e.g., mesh-grooved wicks) balance capillarity and permeability, reducing thermal resistance by up to 48.9% (Xiong et al. [30]).
- Screen mesh wicks: High surface area and design flexibility enable robust capillary pumping, though mechanical degradation under thermal cycling remains a concern. Numerical models (Zhang et al. [27]) validated experimentally show 16% lower thermal resistance in composite mesh wicks.
- Sintered wicks: High thermal conductivity and mechanical strength make them ideal for high-heat applications. However, pore uniformity challenges and energy-intensive fabrication limit scalability. Surface modifications (e.g., nanostructuring) improve hydrophilicity, reducing thermal resistance by 38.1% (Sun et al. [47]).
- Nanoparticle-enhanced wicks: Ultrathin nanoparticle layers (e.g., silica, Cu) enhance capillarity and nucleation sites, achieving 63% lower thermal resistance (Wang et al. [51]). However, long-term nanoparticle stability requires further investigation.

This analysis underscores the necessity of iterative model refinement using experimental data to optimize wick designs for real-world applications.

Current Challenges are:

1. Nanoparticles sedimentation and agglomeration
  - Issue: Nanoparticles in nanofluids tend to settle or cluster over time due to gravitational forces, van der Waals interactions, and insufficient stabilizers (Brusly et al. [50]; Wang et al. [51]). This compromises capillary performance, leading to uneven thermal distribution, localized dry-out, and reduced heat transfer efficiency.
  - Consequences: Experimental studies (Sun et al. [47]; Guo et al. [48]) reveal sedimentation-induced hotspots and diminished thermal conductivity, contradicting idealized model predictions.
  - Gaps: Existing models often neglect time-dependent particle dynamics, oversimplifying nanofluid behavior.
2. Advanced material integration and durability
   Issue: While advanced materials like graphene, carbon nanotubes (e.g., CSs-Ni-Wick [28]) offer superior thermal conductivity, their integration into wicks faces challenges:
  - Compatibility: Mismatched thermal expansion coefficients between materials induce stress fractures.
  - Consequences: High-performing lab-scale designs (e.g., 373 improved thermal conductivity in surface-treated foams [49]) struggle with real-world longevity.
3. Scalability and manufacturing constraints
  - Issue: Energy-intensive sintering processes (Jiang et al. [45]) and precise nanoparticle deposition techniques (Wang et al. [51]) hinder large-scale production.
  - Example: Ultra-thin vapor chambers with sintered wicks achieve 117,734 W/m^2^ in labs but face cost barriers for industrial adoption due to pore uniformity challenges and redox-induced microcracks.
4. Modeling limitations
  - Issue: Current models (e.g., VOF methods [27], Darcy-Brinkman frameworks [37]) inadequately capture (long-term nanoparticle sedimentation, deformation effects (e.g., bending-induced pore collapse [36]), wettability hysteresis in inclined configurations (Tan et al. [34])).

Future research directions are:

1. Stabilization techniques for nanofluids
  - Surface functionalization: Chemically modifying nanoparticles to repel agglomeration (e.g., surfactants, covalent bonding).
  - Dynamic dispersion systems: Integrating ultrasonic or magnetic mechanisms to redisperse settled particles during operation (Guo et al. [48]; Zhang et al. [27]).
2. Hybrid and composite material development
  - Multifunctional wicks: Combining hierarchical porosity (composite wicks [22,23,24]) with nanostructured surfaces (Sun et al. [47]) to synergize capillarity and thermal conductivity.
  - Bio-inspired designs: Mimicking natural structures (e.g., leaf venation, spider silk) for optimized fluid transport (Wang et al. [24]).
3. AI-driven design and optimization
  - Machine learning: Training models on experimental datasets to predict optimal wick geometries, nanoparticle concentrations, and operational parameters (Zhang et al. [27]; Ma et al. [28]).
4. Sustainable and high-performance materials
  - Green nanofluids: Exploring biodegradable nanoparticles (e.g., cellulose-based) to reduce environmental impact.
  - High-temperature alloys: Developing refractory metal wicks (e.g., Mo-Re composites) for aerospace applications (Xiong et al. [25]).
5. Enhanced multiscale modeling
  - Particle-resolved simulations: Incorporating discrete element methods (DEM) to track nanoparticle movement in nanofluids.
  - Deformation-aware models: Coupling mechanical stress analysis with thermal-fluid dynamics to predict wick performance under bending/flattening (Sangpab et al. [44]).

Addressing these challenges requires a multidisciplinary approach, bridging materials science, fluid dynamics, and advanced manufacturing. Future efforts must prioritize translating theoretical advancements—such as AI-optimized wicks and stabilized nanofluids—into scalable, durable solutions. By refining models to account for real-world complexities (e.g., sedimentation, material degradation), researchers can unlock the full potential of nanofluid-enhanced heat pipes in extreme environments, from compact electronics to space exploration.

## 4. Mathematical Conditions for Condensation and Evaporation

Heat pipes are highly efficient heat transfer devices widely used in electronics, aerospace engineering, energy systems, and thermal management applications. Their operation relies on phase-change processes—evaporation and condensation of the working fluid—which enable exceptionally high thermal conductivity with minimal temperature gradients. However, the performance of heat pipes critically depends on accurately modeling evaporation and condensation, as these processes govern the system’s thermal and hydrodynamic behavior.

Mathematical models of evaporation and condensation are essential for predicting heat transfer, determining maximum operating limits, and optimizing heat pipe design (e.g., wick structure, vapor channel geometry, and working fluid selection). These models also help prevent critical failures such as wick dry-out or vapor channel flooding.

Incorrect modeling of phase-change conditions can lead to reduced heat transfer efficiency due to inaccurate heat flux predictions, degradation of the heat pipe (local overheating, wick destruction, material corrosion), operational instability (thermohydraulic oscillations, capillary or boiling limits).

The basic law for the evaporation of pure steam is the Hertz-Knudsen relation [52]. The mass flow during evaporation from the surface of a liquid in the case of an ideal gas can be described by the ratio:

(26) $$J=\gamma \left(\rho_{s}-\rho_{v}\right)\sqrt{\frac{kT}{2\pi m}}$$

or using saturated vapor pressure *P_s_* and vapor pressure near the surface *P_V_*:

(27) $$J=\gamma \left(P_{s}-P_{v}\right)\sqrt{\frac{m}{2\pi kT}}$$

and models based on these relations [53]. The mathematical expression for the condensation condition will be similar, except for the sign for *J* and the difference in density or pressure. This approach is quite common in the literature, in addition, there are more detailed works that take into account various types of coefficients in the Hertz–Knudsen ratio [54,55]. CFD methods are also the main approach in numerical modeling of phase transitions [56,57,58]. Adachi et al. [59] in the loop heat pipes system have calculated amount of evaporation as:

(28) $$m_{ev}h_{iv}=Q_{w}-Q_{HL}$$

and on the out surface of the wick calculated vapor-liquid interface as:

(29) $$T_{\mathrm{int}}=T_{cc}+\frac{T_{cc}\left(\rho_{l}-\rho_{v}\right)}{\rho_{l}\rho_{v}h_{iv}}\left(\Delta P_{tot}-\Delta P_{wick}\right)$$

Tan et al. [60] have improved the model of Lee [61] evaporation-condensation using VOF approach and dynamic modification model. In this model the used *S_m_* parameter can be written as:

For evaporation:

(30) $$S_{m}=-\beta_{e}\alpha_{l}\rho_{l}\frac{T-T_{sat}}{T_{sat}}$$

For condensation:

(31) $$S_{m}=-\beta_{c}\alpha_{v}\rho_{v}\frac{T-T_{sat}}{T_{sat}}$$

The most common numerical methods allow correct modeling of evaporation and condensation processes it is VOF (Volume of Fluid) [62,63] and Level-Set method [64] and combined level set—VOF methods [65,66].

The processes of evaporation and condensation can be described using Lattice Boltzmann Method. Kupershtokh and Alyanov [67] have proposed a numerically efficient method for specifying the steam flow on a flat boundary of the computational domain by calculating distribution functions based on the incoming characteristics of the Lattice Boltzmann Method [67]. Kirillov et al. [68] have proposed special equation for calculation of condensation/evaporation rate *L*:

(32) $$L=\frac{k\Delta p}{\sqrt{2\pi MRT}}$$

where *L* (mol/(m^2^ s)) is the evaporation rate, *k* is the condensation (evaporation) coefficient, Δ*p* (Pa) is the pressure difference, *R* (J/(K mol)) is the gas constant, *M* (kg/mol) is the molecular mass of the evaporant, *T* (K) is the temperature.

Wadekar and Kenning [69] have developed a simplified mechanistic model to describe evaporation heat transfer in vertical slug flow. Although originally formulated for thin-film evaporation, the same approach can be extended to condensation by inverting the temperature gradient driving the phase change. Their model employs a length-weighted average of the heat transfer coefficients in the bubble and slug regions, as expressed in Equation (26). The results have shown a good agreement with experimental data at low flow rates

(33) $$\alpha =\alpha_{f}\frac{L_{b}}{L_{b}+L_{s}}+\alpha_{s}\frac{L_{s}}{L_{b}+L_{s}}$$

Li et al. [70] based on the assumption that the air reached the water temperature at the air–water interface and was completely saturated, have received the ratio:

(34) $$\Delta m_{wv}=\beta \left[x^{\prime }(t_{w})-x\right]\Delta F_{w}$$

Experiments on measuring the condensation coefficient α_c_ have been conducted by Knacke et al. [71] and Nesmejanow et al. [72]. Calculations have been carried out either by measuring the evaporation rate into a vacuum using the Langmuir relation:

(35) $$\tau_{0}=\frac{q_{c}p_{0}}{\sqrt{2\pi mkT}}$$

or by measuring the evaporation rate and vapor pressure p at the interface and calculating α_c_ using the Hertz-Knudsen formula:

(36) $$\tau_{0}=\frac{q_{c}\left(p_{0}-p\right)}{\sqrt{2\pi mkT}}$$

Kucherov and Rikenglaz [73] have discovered possible inaccuracies in measurements of the evaporation coefficient using the classical Hertz-Knudsen relation and proposed its generalization in the form:

(37) $$\tau =\frac{\chi q_{c}\left(p_{0}-p\right)}{\sqrt{2\pi mkT}}$$

Schrage proposed a modification of the Hertz-Knudsen equation in the form [74]:

(38) $$J=\frac{2}{2-q_{c}}\sqrt{\frac{m}{2\pi R}}\left(q_{c}\frac{p_{v}}{\sqrt{T_{v}}}-q_{e}\frac{p_{0}}{\sqrt{T_{0}}}\right)$$

For a curved surface on which phase transition processes occur, an analogue of the Hertz-Knudsen relation has been presented by Marek [75]. A detailed description of obtaining empirical coefficients and making various experimental corrections to the equations for calculating the coefficients of evaporation and condensation and the total flow due to the phase transition has been presented by Davis [76].

Numerical methods for modeling the flows of a two-phase medium are also often used to describe phase transitions such as evaporation and condensation. In this case, the classical model is presented in [77]. Ryskin and Leal [78] have presented a numerical model based on the finite difference method in transformed variables for modeling free-boundary problems. At the same time, in continuation of this work [79], experimental data are presented for various types of bubbles, their formation and transformation [80,81,82,83,84].

Currently, there are many different models and approaches for accounting the phase transitions in various physical processes (energy jump condition, Hertz-Knudsen-Schrage, Kucherov-Rikenglaz, Lee models). Each of them offers distinct advantages for phase-change simulations, balancing physical accuracy, kinetic effects, and computational efficiency, yet face challenges in experimental validation and interfacial physics. The Level Set (LS) method excels in capturing complex interface topologies but struggles with mass conservation, while VOF method ensures mass preservation but lacks precision in curvature estimation, prompting hybrid approaches like CLSVOF for improved performance. Future progress hinges on enhanced experimental data, particularly high-resolution interfacial measurements, to refine model parameters and validate hybrid LS/VOF techniques. Advancements must focus on integrating these methods with turbulence modeling and microscale physics to address real-world applications like microchannel boiling and spray cooling. Ultimately, the development of universal, high-fidelity CFD tools requires tighter coupling between advanced interface tracking, accurate phase-change models, and robust experimental benchmarks to tackle emerging thermal management challenges.

## 5. Numerical and Experimental Results Overview

**Figure 12:**
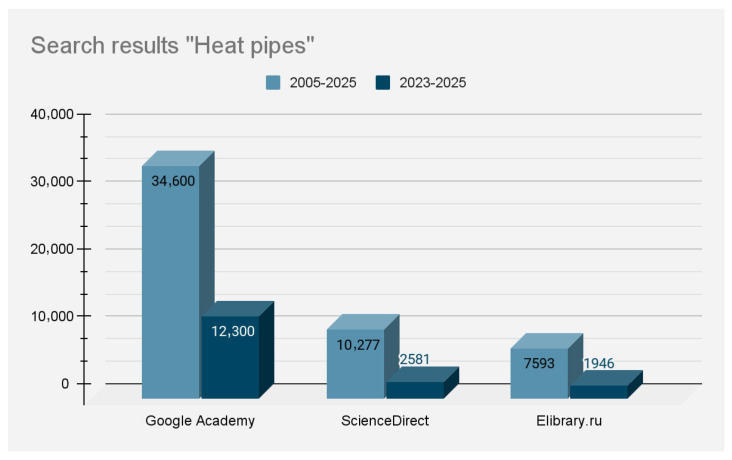
Results of search queries in three scientific databases for the periods from 2005 to 2025 and for the last three years.

In the last twenty years, many research papers have been created for describing different HP systems and modelling them. Amount of searching results of topic “heat pipes” for the considered period is shown in Figure 12.

In this part of review, we demonstrate results of two most useful strategies of researching HP: numerical simulations and experiments.

### 5.1. Numerical Simulations Results

The paper [85] presents a simplified mathematical model for pulsating heat pipes (PHP) aimed at improving heat transfer management in microelectronic applications. The authors utilize the firefly algorithm for efficient parameter estimation from limited experimental data, demonstrating its effectiveness in obtaining key parameters. The paper [86] develops a mathematical model for bent-flattened heat pipes using the “Receding and Excess Fluid” (REF) model to predict thermal resistance and wall temperature. It highlights that bending damages the wick, leading to unsaturation and reduced thermal performance. The study concludes that thermal resistance increases with bending angle due to pressure drops and wick damage. The model’s predictions align well with experimental results, demonstrating its accuracy in assessing heat transfer performance. The study [87] concludes that the heat and mass transfer in a flat heat pipe with an idealized porous wick can be effectively modeled using one-dimensional assumptions, as the axial variations are significantly larger than the pore radius. The mass evaporation rate is primarily dependent on the pipe temperature and is largely independent of vapor pressure differences, leading to the pipe functioning like a fin with evaporative cooling. The research provides analytic solutions for various parameters, including heat rate and flow rates, and offers design guidelines for optimizing flat heat pipes by varying pipe length and wick thickness. Rathore et al. [88] have investigated the phase change and heat/mass transfer in a FPHP-like system using a three-dimensional pore-scale simulation approach. The effective thermal conductivity of the system was found to be around 10,000 W/m-K. The paper [89] deals with numerical simulation of the heat transfer performance of ultra-thin flat heat pipes with copper mesh wick for various heating powers. The temperature patterns and flow properties of ultra-thin flat heat pipes have been studied using the coupling porous media approach and user-defined function (UDF) in FLUENT software. The authors have revealed that a rise of the heating power leads to an increment of the temperature drop of ultra-thin flat heat pipes, whilst the pressure difference and the fluid velocity within the wick are improved. Chhokar et al. [90] have presented a compact mathematical model that predicts the thermal and hydrodynamic performance of a flat micro heat pipe with a rectangular grooved wick. The model utilizes analytical solutions for energy equations and an efficient iterative method, allowing for quick computations on standard workstations. Parametric studies indicate that varying the wetting angle can enhance the maximum heat transfer rate by approximately 15%, while adjusting groove dimensions can increase it by about 20%.

The study [91] has concluded that nanoparticles enhance heat transfer capabilities in screen mesh wick heat pipes when using nanofluids. A two-dimensional transient numerical model predicts vapor core, wall temperatures, and velocities in a screen mesh wick heat pipe. Cu-water nanofluids show a 20% increase in vapor velocity compared to de-ionized water at 350 W. Vijayakumar et al. [92] have investigated the thermal performance of heat pipes using nanofluids, which are suspensions of nanoparticles in heat transfer fluids. A maximum decrease in total thermal resistance was observed, with values of 83% for Al_2_O_3_, 79% for CuO, and 76% for TiO_2_ nanoparticles. The optimal nanoparticle concentrations were determined to be 25% by volume for Al_2_O_3_ and TiO_2_, and 35% for CuO, which correspond to the capillary limit for effective heat transfer. The paper [93] has investigated the thermal performance of heat pipes using nanofluids, focusing on the effects of nanoparticle aggregation and deposition on thermal properties and capillary limits. The optimal concentration of Al_2_O_3_ nanoparticles for enhancing capillary limit and reducing thermal resistance was determined to be 0.5% w/w. Experimental and numerical results indicated that higher heat input was required to reach the capillary limit with nanoparticles, but excessive concentrations led to increased thermal resistance. In 2022, a review paper [94] has published entirely devoted to describing the features of numerical modeling of heat pipes used in various devices and systems. The paper describes various types of heat pipes, such as thermosyphon heat pipe, variable conductance heat pipe (VCHP), loop heat pipe (LHP), pulsating/oscillating heat pipe (PHP/OHP). Mahjoub and Mahtabroshan [95] have demonstrated the construction of a mathematical model based on the Navier-Stokes equations and Darcy’s law, as well as the use of SIMPLE algorithm to evaluate the heat transfer process in a two-dimensional formulation. Researchers [96] using special French CASCO software (French abbreviation for Code Avancé de Simulation du Caloduc Oscillant) have demonstrated features of the heat pipe operation associated with bubble generation. Other interesting results of numerical simulation have been obtained in [97], where authors have used ANSYS CFX for HP modeling. Model of PHP has been the object of investigation in [98] by using C++ language and OpenFOAM. Programming language FORTRAN 90 and Finite Volume Method have been used in [99] for capillary pumped loops (CPL) and loop heat pipes (LHPs) modelling. Lattice Boltzmann Method (LBM) and OpenMP parallel algorithm for C++ code of numerical simulation presented in [100]. Ling et al. [101] have used Matlab R2016b to realize the microchannel separate heat pipe (MCSHP). Table 3 structuring the part of the review devoted to the consideration of the results of numerical simulation.

It is worth noting separately the increasing use of machine learning methods and other AI-based approaches to study the properties of heat pipes. Some results of such use are presented in [102,103,104].

### 5.2. Experimental Results

Lee et al. [105] have presented a concentric annular heat pipe (CAHP) to enhance heat transfer using distilled water as a working fluid. Experiments and one-dimensional analysis were conducted to predict thermal characteristics and evaluate performance. The CAHP achieved a thermal resistance of 3.74 °C/W under specific conditions. One-dimensional simulation model using AMESIM has been verified against experimental results. The experimental investigation [106] has concluded that the heat pipe (HP) outperforms both the two-phase closed thermosyphon (TPCT) and the thermosyphon heat transport device (THTD) under isothermal conditions, with HP exhibiting a minimal temperature gradient. The thermal resistance (TR) of TPCT and THTD is significantly higher than that of HP, with THTD performing better in the 50–80 °C range but deteriorating above 80 °C due to boiling. THTD is recommended for long-distance heat transport applications due to its consistent TR, while TPCT is less effective as transport distance increases. An important type of heat pipe is the ultrathin heat pipe, researches [107,108,109,110,111] have performed the experimental works, while authors [30,112,113,114,115] have received the simulation results. Moreover, we can observe fresh results from work [116]. The paper [116] has developed three types of composite wicks for UTHPs with a 0.6 mm thickness: copper foam and mesh wick (CFMW), two layers of different mesh wick (TDMW), and three layers of the same mesh wick (TSMW). The startup and steady-state performances of the UTHPs with liquid filling ratios of 60% to 120% have been studied. It has been found that the CFMW UTHP with a filling ratio of 100% exhibited the best startup performance, with the highest equilibrium temperature of 58.37 °C. Zhang et al. [117] have discussed experimentally the parallel microchannel loop heat pipe (PMLHP) and the self-similar fractal microchannel loop heat pipe (SFMLHP) highlighting the aspects of filling ratio and inclined angle. Experimental results have shown that PMLHP had four operation phenomena similar to the dendritic bionic microchannel loop heat pipe (DBMLHP) whilst SFMLHP only had two operation phenomena. Lu et al. [118] have investigated the thermal performance of gravity heat pipes (GHP) with various pipe configurations, including vertical, oblique, and curved designs. The study establishes mathematical correlations for key performance metrics, enhancing understanding of GHP thermal-hydraulic mechanisms and guiding future design improvements. The optimal configuration identified was Type E (curved GHP with a curving angle of 30), achieving the highest heat transfer efficiency of 73.1 and the lowest thermal resistance of 0.37 CW. The study [119] has compared wall temperature profiles and thermal resistance with de-ionized (DI) water. The effects of nanoparticle volume fraction and size on thermal performance are analyzed. The thermal performance can be enhanced by about 100% with less than 1.0 vol% of nanoparticles, and thermal resistance decreases with increasing nanoparticle size. Other work [120] of the same researchers has investigated the thermal performance of circular screen mesh wick heat pipes using water-based Al_2_O_3_ nanofluids at volume fractions of 1.0 and 3.0 Vol.% compared to DI water. Results have shown that the average evaporator wall temperatures are significantly lower with nanofluids, and thermal resistance is reduced by approximately 40% at the evaporator-adiabatic section. The maximum heat transport rate of the heat pipes is improved with the use of nanofluids. Putra et al. [121] have concluded that nanofluids enhance thermal performance in screen mesh wick heat pipes compared to conventional fluids. The research determined that the screen mesh wick heat pipe with Al_2_O_3_-water nanofluid at a 5% volume concentration exhibited the best thermal performance. The study [122] has concluded that the use of silver nanoparticles in de-ionized water significantly enhances the thermal performance of heat pipes. The study demonstrates a significant improvement in the thermal performance of heat pipes using silver nanoparticles in De-ionized water, with a substantial reduction in thermal resistance of 76.2% observed at a 0.009 vol. concentration of silver nanoparticles. An enhancement in the evaporation heat transfer coefficient of 52.7% is also noted for the same concentration. Vijayakumar et al. [92] have investigated the thermal performance of heat pipes using nanofluids, which are suspensions of nanoparticles in heat transfer fluids. A maximum decrease in total thermal resistance was observed, with values of 83% for Al_2_O_3_, 79% for CuO, and 76% for TiO_2_ nanoparticles. The optimal nanoparticle concentrations have been determined to be 25% by volume for Al_2_O_3_ and TiO_2_, and 35% for CuO, which correspond to the capillary limit for effective heat transfer.

Good review paper [123] at 2013 compiles results from 38 experimental studies and 4 modeling approaches. The paper has reviewed advancements in the application of nanofluids in thermosyphons, heat pipes, and oscillating heat pipes, focusing on the effects of nanoparticles on thermal performance. At 2018, Australian research group, has presented the research [124] focusing on the advancements and applications of heat pipe solar collectors (HPSCs) in various fields, including domestic water heating, desalination, space heating, and power generation. They have observed 182 papers, identified challenges, and research gaps in the field, suggesting future research directions for improving HPSCs in applications such as water heating, desalination, space heating, and electricity generation. The paper [125] investigates the thermal performance of screen mesh wick heat pipes using water-based copper nanofluids, highlighting a thermal conductivity enhancement of approximately 15% at 30 °C with 0.5 wt% Cu nanoparticles. The thermal resistance of the heat pipe is reduced by approximately 27% when using 0.5 wt% Cu nanofluid. Kumaresan et al. [126] have presented a comparative study on the thermal performance of sintered wick heat pipes (SWHP) and mesh wick heat pipes (MWHP) using CuO nanofluids. SWHP exhibits a 14.3 times greater heat transport capacity compared to MWHP under identical operating conditions. A significant reduction in surface temperature of 27.08 °C is noted for SWHP with a 1.0 wt.% concentration of CuO nanofluid. Next work of that research group [127] has studied the enhancement of heat transfer in a copper sintered wick heat pipe using surfactant-free CuO nanoparticles in DI water. A temperature difference of 5.1 °C is noted between the surface and vapor core at the evaporation section. Thermal resistance is reduced by 66.1% for 1.0 wt.% CuO nanofluid compared to DI water. The paper [128] has investigated the thermal performance enhancement of variable conductance heat pipes using water-based CuO-nanofluids at volume fractions of 1, 3, and 5 Vol.% and varying amounts of non-condensable gas (air). The maximum improvement in thermal resistance achieved with the water-based CuO-nanofluid is 9.5% at a coolant temperature of 18.3 °C and a non-condensable gas mass of 0.5 mg. The study [129] has investigated biomaterials as wicks and nanofluids as working fluids in loop heat pipes. Loop heat pipes (LHPs) are efficient for electronic cooling, utilizing capillary forces for fluid circulation. The biomaterial Tabulate wick demonstrated a 56.3% reduction in thermal resistance compared to sintered powder wicks. The optimal thermal resistance was observed with a biomaterial wick combined with Al_2_O_3_-water nanofluid as the working fluid. Chiang et al. [130] have investigated the effects of wick structures and working fluids on thermal resistance and critical heat flux in heat pipes. It was found that microstructures of grooved or sintered wicks significantly reduced thermal resistance (R), while sintered wicks also improved critical heat flux (CHF). Low concentrations of MNFs enhanced R reductions and promoted CHFs more effectively than sintered wicks. Strong magnetic fields had minimal impact on R reduction but could enhance performance when combined with high MNF concentrations. The study [131] has investigated the heat transfer characteristics of inclined copper sintered wick heat pipes using surfactant-free CuO and Al_2_O_3_ nanofluids. The minimal temperature difference between the surface and vapor in the evaporator section was observed, indicating improved heat transfer efficiency. The thermal efficiency reached a maximum of 30.42% for CuO nanofluids and 26.17% for Al_2_O_3_ nanofluids at their respective optimal concentrations. The paper [51] has investigated the use of a silica nanoparticle layer as a thin wick in heat pipes and vapor chambers to enhance thermal performance for portable electronic systems. The nanoparticle layer on the copper tube inner wall can function as a thin wick for heat pipes and vapor chambers. Replacing distilled water with nanofluid improved thermal resistance and maximum heat transfer rate. The nanoparticle layer allows heat transfer even without the original wick, reducing thermal resistance. The maximum heat transfer rate was slightly less than the original heat pipe but comparable. The silica nanoparticle layer improved thermal resistance from 0.62 to 0.23 K/W compared to wire mesh wicks. Weng et al. [132] have investigated the heat transfer performance of a pulsating heat pipe (PHP) using three working fluids: ultrapure water, n-butanol self-rewetting fluid (SRWF), and self-rewetting Fe_3_O_4_-nanofluid (SRNF) under varying conditions of filling ratio (FR), heating power, and magnetic field. At FR = 50%, the overall thermal performance was optimal, with thermal resistance reductions of 14.5–27.8% for self-rewetting fluids compared to ultrapure water. The effective heat transfer coefficient for SRNF-PHP reached 5967.6 W/m·K, approximately 13.92 times greater than that of copper. The paper [133] has investigated the influence of various kinds of wicks and working conditions on the time-dependent thermal behavior of heat pipes. The authors have ascertained that the thermal resistance is decreased from 0.6 K/W (at 25 W) to 0.05 K/W (at 200 W) by rising the heat pipe diameter from 6 to 10 mm. Dhairiyasamy and Gabiriel [134] have investigated the thermal performance of heat pipes using hybrid nanofluids composed of multi-walled carbon nanotubes (MWCNT) and aluminum oxide (Al_2_O_3_) nanoparticles. The study has concluded that hybrid nanofluids composed of MWCNT and Al_2_O_3_ significantly enhance the thermal performance of heat pipes, achieving a minimum thermal resistance of 0.80 K/W and a peak heat transfer coefficient (HTC) of 2250 W/m^2^·K at optimal conditions (90% filling ratio, 90° inclination angle, and 80 W heat input). The research indicates a 40% improvement in HTC compared to conventional fluids and highlights the importance of nanoparticle concentration in optimizing heat transfer efficiency. The paper [135] has investigated a three-turn pulsating heat pipe using nanofluids for efficient heat dissipation in microelectronics. The study compares heat transfer performances of nanofluids with deionized water, highlighting superior characteristics of Graphene/H_2_O. Graphene/H_2_O shows the best heat transfer performance at 1.0 wt% concentration, 80% filling ratio, and 60° tilt angle.

A large number of works are devoted to the production and experimental verification of various hypotheses related to the design features of heat pipes. Table 4 contains some structured information on existing works in this direction.

Table 5 shows major results of some paper devoted to heat pipes.

In conclusion of this section, it is worth noting that comparing the presented works with an emphasis on numerical modeling, one can note a certain trend towards complication of both the numerical models themselves and a more extensive use of the resources provided by modern computing technology. At the same time, classical methods are still widely represented. In the case of experimental studies, unfortunately, most researchers are limited to commercial heat pipes, while the widespread creation of experimental versions of unique heat pipe configurations, in terms of the working fluid used, in terms of work related to nanofluids, is positive. Another important point is that a significant part of the research groups provides both numerical modeling data and their own experimental data, which is convenient material for further work by other researchers. Speaking about the existing difficulties, it is worth noting the separate complexity of experimental studies in laboratory conditions of various extreme operating modes of special heat pipes (microgravity, nuclear power, nanoscale devices) and problems associated with the limitation of the measuring methods used. Touching upon the difficulties associated with computational experiments, one cannot help but note the difficulty of taking into account the real geometry and defects of materials, the numerical implementation of multiphase and nonlinear processes, as well as the computational complexity of these problems. A separate problem for numerical studies is the lack of experimental data for validation.

## 6. Heat Pipes Applications

Most often, heat pipes can be used for microelectronics and laser devices cooling, spacecraft heat change, permafrost cooling, solar and geothermal system construction and some more specific areas. In this article we collected examples of application HP systems for different areas. Researchers working with heat pipes as an object of study, starting with such classics as Grover [147] and up to modern researchers including Faghri [138], Maydanik and Chernysheva [148], Werner et al. [149], Choi and Zhang [150] and others, distinguish the following types of heat pipes, which we have tried to present in Table 6 and Table 7.

For a more detailed consideration of the possibilities of using heat pipes, let us consider their individual use cases in more detail.

### 6.1. Spacecraft Heat Change

**Figure 13:**
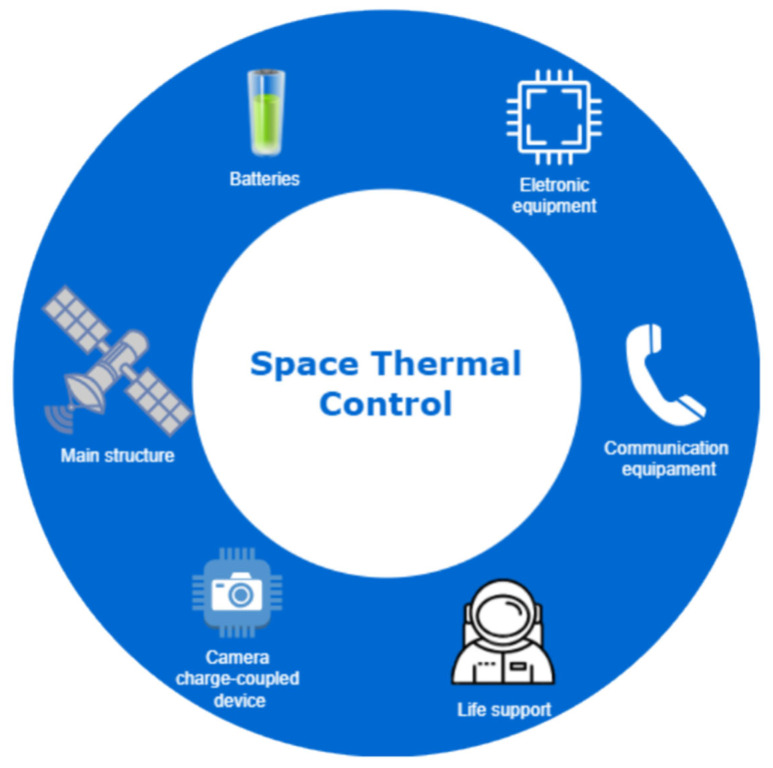
Equipment that needs thermal control in space. Reproduced from ([176]). Open Access Article.

The paper [167] has presented a loop heat pipe (LHP) designed for space missions, focusing on miniaturization and alternative working fluids. An experimental LHP was tested with acetone, managing up to 70 W of heat. The proposed LHP is deemed reliable for future space missions, addressing concerns about hazardous working fluids. A comprehensive survey [168] and analysis of space missions utilizing CHL technologies were presented, highlighting the need for advanced mathematical modeling to improve performance predictions. The challenges in generating precise mathematical models for two-phase capillary pumped devices were acknowledged, emphasizing the complexity of the physical phenomena involved. The development of numerical modeling technology for loop heat pipes (LHP) has been initiated, indicating ongoing advancements in this field. Jie et al. [169] have concluded that low-cost mass or power control is essential for two-phase thermal control systems in space applications. The design incorporates thermoelectric coolers (TECs) to enhance refrigeration capacity and control accuracy. The experiments demonstrated the system’s robustness and reliability under various conditions, with effective temperature management for a heat load of up to 200 W using CO_2_ as the working fluid. The paper [170] has investigated the processing of nickel-based sintered porous wicks for use in capillary pumped loops (CPL) and loop heat pipes (LHP) for spacecraft thermal management. It emphasizes the importance of achieving high porosity, small pore size (less than 5 µm), and high permeability (greater than 10 m-Darcy) to enhance capillary action and fluid transport. Experimental trials involved sintering carbonyl nickel powders at various temperatures to optimize these properties, with successful results yielding a cylindrical wick with 64% porosity and 70 m-Darcy permeability. Yunze et al. [171] have proposed a dual-driven intelligent combination control strategy for spacecraft thermal management. It improves temperature control and heat flux tracking through adaptive fuzzy fusing of control actions. A four-nodal mathematical model analyzes the dynamic characteristics of the heat pipe cooling system. The paper [172] has proposed a fuzzy incremental control strategy for LHP space cooling systems to enhance thermal control reliability. It presents a mathematical model for dynamic analysis and evaluates control schemes numerically. The findings aim to improve thermal control in advanced spacecraft, including nano-satellites and Mars landers. Kim et al. [173] have presented a new thermal control hardware combining heat pipes (HP) and phase change materials (PCM) for high heat dissipation components. They develop thermal models for numerical analysis of a honeycomb structure radiator with HP and PCM. The obtained results can be used for constructing spacecraft thermal control hardware. The paper [174] investigates effective thermal conductivity (ETC) in bi-porous sintered structures, particularly focusing on loop heat pipes (LHPs) used in spacecraft and satellites. It compares analytical and numerical methods for predicting ETC, utilizing thermal resistance network theory and the Finite Volume Method (FVM) with a 3D porous structure. The study finds that while both methods yield congruent results for fine nickel powder samples, coarse samples show discrepancies due to irregular particle shapes. Miranda et al. [175] have investigated the resilience of Variable Conductance Heat Pipes (VCHP) under prolonged exposure to subfreezing temperatures, specifically focusing on the detection of frozen ammonia blockages in the inactive section of the heat pipe. The study demonstrated that the temperature distribution along the VCHP is crucial for understanding the formation of frozen ammonia blockages, which can lead to malfunctioning. The results indicate a strong correlation between adiabatic section and reservoir temperatures during blockage occurrences. In 2024, a review paper [176] has been published on heat exchange in outer space (see Figure 13).

It examined the results of 103 scientific articles on the topic and presented interesting results. The review concludes that two-phase cooling tests in microgravity exhibit lower performance compared to those conducted on Earth due to the absence of buoyancy forces affecting surface tension and other properties. The study also notes advancements in fluid modeling, although a margin of error of around 20% or higher remains, complicating simulations in microgravity conditions.

### 6.2. Microelectronics Cooling

**Figure 14:**
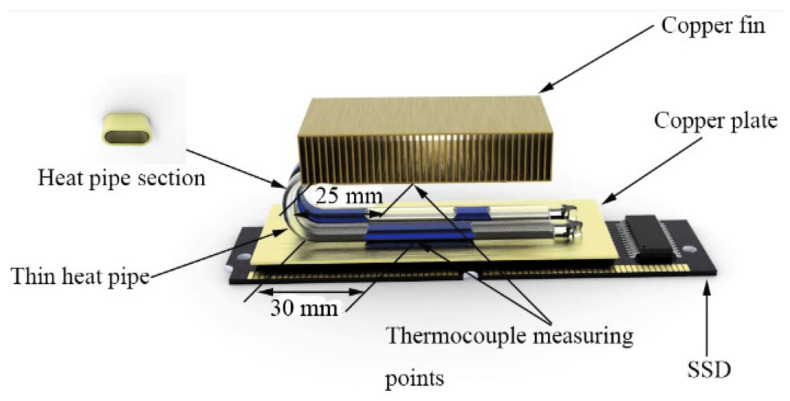
Schematic diagram of THP heat sink for cooling of SSDs. Reproduced from ([181]). Open Access Article.

Micro heat pipes usually can be used in microelectronics cooling. The study [177] concludes that heat transfer and fluid flow in polygonal and rectangular micro heat pipes can be effectively modeled, revealing that heat transfer occurs through evaporative vapor flow and conduction in liquid-filled corners and pipe walls. It models various polygonal micro heat pipes and finds that their heat transfer coefficients are lower than conventional heat pipes. The study derives analytic solutions for temperature profiles and pressure distributions, revealing that evaporation occurs mainly in a boundary layer at the contact line. The review [178] presents recent advances in various MEMS-based micro heat pipes (MHPs), including micro-GHP, micro-CPL, micro-LHP, micro-OHP, and micro-VC, highlighting the significance of flexible polymer heat pipes for electronics integration. Experimental results indicate that micro capillary pumped loops (CPLs) can achieve heat fluxes up to 200 W/cm^2^, demonstrating effective cooling for high heat flux devices. The paper [179] discusses ultra-thin heat pipes (UTHPs) as a solution for efficient heat dissipation in microelectronics. The study concludes that increasing the mesh number enhances the start-up performance and reduces thermal resistance in steady-state operation of ultra-thin flattened heat pipes (UTHPs). However, excessively high mesh numbers can lead to decreased permeability and increased flow resistance. The tilt angle significantly affects heat transfer performance, with higher angles improving maximum heat transfer capacity and evaporator temperature. Wu et al. [180] have identified three operation modes in pulsating heat pipes: small pulsations, bulk pulsations, and circulation, which can exist independently or coexist with increased heat input. It concludes that the pulsation in the 2 mm heat pipe is more vigorous than in the 1 mm heat pipe due to higher pulsating frequency and amplitude. These results can be used in new design of microelectronic cooling systems. The study [181] concludes that the application of thin heat pipes (THPs) significantly improves the heat transfer performance of solid-state drives (SSDs) to address overheating issues (see Figure 14).

The heat transfer process involves three stages: linear temperature rises, nonlinear temperature rises, and a stable stage, with a startup time of about 90 s for a single THP at 50 W input. Experimental and numerical results showed a maximum temperature difference of 3.53 °C between observed and simulated values, confirming the reliability of the findings.

### 6.3. Solar and Geothermal System Construction

**Figure 15:**
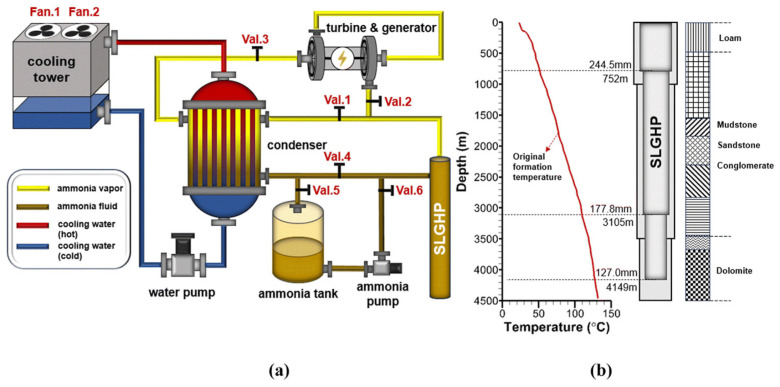
(**a**) super-long gravity heat pipes super-long gravity heat pipes (SLGHP) geothermal system constructed in Xiong’an, China; (**b**) measured temperature in the original geothermal formation, well-borehole configuration after assembly with the SLGHP, and local geological formation [188]. Reproduced from ([188]). Open Access Article.

The one of the most important problems is global warming and many researchers’ teams are trying to find the way to solve this big problem of humanity. Heat pipes can be part of complex solution of this situation. Ming et al. [182] have discussed the urgent need to address global warming, emphasizing that reducing anthropogenic greenhouse gas emissions is essential but challenging due to economic reliance on fossil fuels. It explores various geoengineering techniques, particularly solar radiation management (SRM) and earth radiation management (ERM), which aim to enhance outgoing longwave radiation and cool the Earth. Chapter 9 “Transferring surface sensible heat to the troposphere” proposes engineering options for removing heat from the Earth’s surface using heat pipes. In 2013, a conference on a similar topic was held in the Freiburg, Germany; work [183] related to the topic of our article deserves special attention. The paper discusses optimizing solar collectors through an integrated, holistic development approach to enhance efficiency and reduce costs. Innovative holistic heat pipe facade collector concepts are being developed, focusing on maximizing efficiency, minimizing costs, and ensuring aesthetic integration to enhance acceptance by architects and society. Morawietz et al. [184] have presented operating limit measurements for a self-fabricated and a commercial solar heat pipe at horizontal and small inclination angles. The commercial solar heat pipe showed good reproducibility in operating limit measurements, unlike the self-fabricated one, which varied drastically. Föste et al. [185] have investigated the use of butane heat pipes in solar thermal collectors to reduce stagnation temperature loads, demonstrating their effectiveness in maintaining high collector performance at temperatures up to 120 °C. Experimental results indicate that the thermal conductance of butane heat pipes can be significantly improved by increasing the condenser area and incorporating helical grooves on the inner surface, leading to more than double the thermal conductance compared to standard designs. This enhancement allows for better performance of solar thermal collectors, preventing evaporation of the solar fluid under typical operating pressures. The study [186] has investigated the thermal performance of a serpentine thermosyphon heat pipe solar collector under real operating conditions. Various working fluids, including water and Al_2_O_3_ nanofluids, were tested for performance. The effects of coolant rate, nanomaterial concentration, and tilt angles on performance were analyzed. The experiments demonstrated that the thermal performance of the serpentine shape thermosyphon heat pipe flat plate solar collector improved with the use of nanofluids, specifically Al_2_O_3_-water, compared to pure water. The enhancement in performance was quantified, showing increases of 3.79%, 10.72%, and 15.24% for concentrations of 0.05, 0.25, and 0.5 wt%, respectively. Kim et al. [187] have presented the development and demonstration of a hybrid solar geothermal heat pump polygeneration system, modeled and simulated in TRNSYS, and installed in Cheongju, Korea. The system effectively meets the building’s heating and cooling loads of 13.8 kW and 10.6 kW, respectively, under specified ambient temperatures. Huang et al. [188] have shown the development of a super-long gravity heat pipe (SLGHP) designed to exploit deep geothermal energy, achieving a length of 4149 m. The SLGHP system, utilizing ammonia as the working fluid, demonstrated continuous heat output exceeding 1 MW and successfully generated electricity at a rate of approximately 7 kW during testing (see Figure 15). The study concludes that conventional geothermal energy extraction technologies face significant drawbacks, including environmental issues and slow commercialization of Enhanced Geothermal Systems (EGS).

### 6.4. Interdisciplinary Research and Heat Pipes Improving

Shen and Guo [189] have investigated the design and analysis of a biomimetic lattice structure for use as a wick in heat pipes, inspired by plant transport systems. The biomimetic lattice demonstrated superior capillary performance, with K/r eff parameters significantly higher than traditional structures. The research analyzed six lattice configurations (SC, BCC, FCC, BCCZ, FCCZ, FCHC) across four porosity levels (40%, 50%, 60%, 70%). The FCHC lattice consistently outperformed others in capillary performance at all porosity levels, indicating its potential for optimized heat pipe applications.

## 7. Future Research

Taking into account the performed analysis of the transport phenomena in heat pipes from mathematical and physical points of view it is possible highlight the future research for this topic:

- Development of mathematical models to describe in detail the transport phenomena for heat pipes with nanofluid as a working fluid, where it is necessary to take into account the nanoparticles aggregation, stability of the nanofluid, changes in surface wettability, or the formation of nanolayers during evaporation/condensation.
- More detailed simulation of the wick porous structure taking into account the heterogeneity of the porous wick.
- Research in hybrid and composite materials to create the effective porous wick.
- Development of AI-driven design and optimization not only for simulation of transport processes but also for creation of optimal heat pipes.
- Creation of effective nanofluid using green nanoparticles and effective base fluids.
- Development of numerical and analytical techniques for effective simulation of fluid flow, heat and mass transfer in heat pipes.

## 8. Conclusions

This report provides a comprehensive overview of the researches on mathematical description of transport phenomena in heat pipes filled with nanofluid taking into account special conditions for condensation and evaporation zones. According to the physical side of the problem, analysis of wicks structure and transport phenomena in wicks has been described. Additionally numerical and experimental results combined with applications of heat pipes has been studied. The following major points are made considering the present review:

- Nowadays, there are many different correlations for the physical properties of nanofluids that can be used as working fluids in heat pipes. More important to use the experimentally-based correlations with single-phase nanofluid models or it is possible to use the theoretical correlations but combined with two-phase nanofluid models.
- Description of transport phenomena in wicks can be performed also using the average approaches based on the porous medium models, but it is possible to design the structure of the porous material from practice.
- Applications of heat pipes can be found in various practical fields including building engineering, power engineering, electronics and medicine engineering, where the used heat pipes can be improved with more effective working fluid or optimal structure of the wick combined with condensation/evaporation sections.
- As a result, the future research can be focused on unifying theoretical, experimental, and computational approaches to optimize nanofluid selection and wick design for practical applications.
- From another side, a development of numerical approaches for solution of boundary-value problems for fluid flow, heat and mass transfer in heat pipes is very important also.

## Figures and Tables

**Table 1:** Heat transfer performance, pressure drop and stability of wick structures.

Wick Type	Heat Transfer Performance	Pressure Drop Characteristics	Stability/Limitations	Key Studies
Composite (porous) wicks	- 16.7% higher heat dissipation (segmented designs) - Thermal resistance: 0.069–0.79 °C/W	- Reduced interfacial flow resistance - Optimized vapor-liquid segregation	- Susceptible to clogging in contaminated environments - Complex fabrication	Yi et al. [22,23]; Wang et al. [24]
Grooved wicks	- 48.9% lower thermal resistance (hybrid designs) - Effective in low-gravity conditions	- Low hydraulic resistance - 25.1% vapor velocity reduction	- Dry-out under high heat fluxes - Geometry-dependent performance	Xiong et al. [30]; Wang et al. [32]
Screen mesh wicks	- Thermal resistance: 0.023–0.042 °C/W - High surface area for capillary action	- Permeability depends on mesh layers (A/B/C configurations) - <10% prediction error	- Mechanical degradation under thermal cycling - Limited pore size control	Tan et al. [34]; Nookaraju et al. [39]
Sintered wicks	- 38.1% lower thermal resistance (nanostructured) - Capillary pressure: 0.65 Pa	- High flow resistance in deformed wicks - 155% thermal resistance increase if bent	- Energy-intensive manufacturing - Pore uniformity challenges	Sangpab et al. [44]; Ginting et al. [46]
Nanoparticle-enhanced	- 63% thermal resistance reduction (silica layer) - Enhanced nucleation sites	- Increased surface roughness elevates condenser resistance	- Nanoparticle sedimentation/agglomeration - Coating instability under prolonged use	Brusly et al. [50]; Wang et al. [51]

**Table 2:** Heat Transfer Performance, Pressure Drop, and Stability of Nanofluids.

Nanofluid Type	Thermal Conductivity Enhancement	Impact on Stability	Key Applications	Key Studies
Cu nanoparticles	- 40% evaporator resistance reduction - Improved boiling efficiency	- Sedimentation-induced hotspots - Requires surfactants for stabilization	Electronics cooling, aerospace	Brusly et al. [50]
Al_2_O_3_ nanoparticles	- 10–30% higher thermal conductivity - Reduced thermal gradients	- Agglomeration under high heat - Wettability hysteresis	Solar collectors, industrial cooling	Sun et al. [47]
Carbon nanotubes	- 373× effective thermal conductivity (surface-treated foams)	- Degradation under prolonged high heat - High cost	High-power electronics, aerospace	Yang et al. [49]
Self-rewetting fluids	- 53.6% higher evaporator coefficients - 66% temperature uniformity improvement	- Sustained performance under thermal gradients - Non-linear surface tension dynamics	Gravity heat pipes, renewable energy systems	Guo et al. [48]

**Table 3:** Analysis of HP types considered in the work with a focus on the features of numerical methods.

Heat Pipe Structure	Reason/Goal	Methods Used	Reference
simplified HP model	Optimization of HP parameters	Firefly algorithm Finite Differences Method FDM	[85]
simplified HP model	To evaluate the effect of Al_2_O_3_/water nanofluid concentration on the capillary limit	a numerical model coupling hydrodynamical equations with a population balance for nanoparticle agglomeration and deposition	[93]
cylindrical HP	To study the effect of Cu-water nanofluid on heat transfer performance	A two-dimensional transient analysis model	[91]
cylindrical HP	To analyze parameters affecting heat pipe operation using a numerical model	FLUENT software SIMPLE algorithm	[95]
flat HP	To optimize heat transfer by varying pipe length or wick thickness	Analytic solutions	[87]
flat HP	Compact model for predicting thermal and hydrodynamic performance	Analytic solutions	[90]
flat plate HP	Phase change phenomena in systems analogous to heat pipes using pore-scale numerical simulation	Ansys^®^ Fluent (version 19.0) Volume of Fluid (VOF) method	[88]
flat micro HP	The effects of unsaturated flow in porous media	Volume of Fluid (VOF) method The Pressure-Implicit with Splitting of Operators (PISO) algorithm	[27]
bent-flattened sintered-grooved miniature HP	Impact of wick damage on liquid return and thermal performance	Receding and Excess Fluid (REF) FEM	[86]
micro channel separate HP	to investigate the thermal-hydraulic mechanism and improve the design and operation of the separate HP	Matlab R2016b A finite element method (FEM)	[101]
pulsating HP	To simulate the start-up, functioning, and stopping of multi-branch pulsating heat pipes	in-house CASCO software film evaporation/condensation model (FEC)	[96]
pulsating HP	The heat transfer performance of a pulsating heat pipe using methanol	ANSYS CFX	[97]
pulsating HP	To numerically model a two-dimensional multiphase flow in a pulsating heat pipe	OpenFOAM volume of fluid (VOF) method	[98]
loop HP	To analyze heat and mass transfer with phase change in an evaporator unit cell using a mixed pore network model	code in Fortran90 finite volume method	[99]
loop HP	to numerically simulate evaporation heat transfer in a loop heat pipe	Lattice Boltzmann Method (LBM) OpenMP parallel algorithm for C++ code	[100]

**Table 4:** Some features of the experimental works presented in this section.

Type of HP	Working Fluid	Materials	Reference
heat pipe (HP)	water	Container—copper Screen mesh—phosphor bronze	[106]
heat pipe (HP)	water-based or ethylene glycol-based Al_2_O_3_ or TiO_2_ or ZnO nanofluid	screen mesh wick—stainless steel tube—copper	[121,136]
heat pipe (HP)	de-ionized water silver nanoparticles	copper	[122,137]
heat pipe (HP)	distilled water copper nanoparticles	not specified	[125]
heat pipe (HP)	water-based CuO-nanofluid	copper	[128]
heat pipe (HP)	de-ionized water CuO, Al_2_O_3_ nanofluid	copper	[131]
heat pipe (HP)	multi-walled carbon nanotubes (MWCNT) and Al_2_O_3_ nanoparticles	copper	[134]
flat heat pipe (FHP)	Water with nanoparticles Cu, CuO, Al_2_O_3_	copper	[113]
flat micro-heat pipe (FMHP)	water-based Al_2_O_3_ nanofluid	copper	[119]
ultra-thin flat heat pipe (UFHP)	de-ionized water	Copper foil Copper wick or composite wick with different structures	[40,107,108,109,110,111,112,114,115]
loop heat pipe (LHP)	not specified	carbon spheres modified nickel wick	[28]
loop heat pipe (LHP)	water-based Al_2_O_3_ nanofluid	biomaterial (Collar) as a wick	[129]
gravity heat pipe (GHP)	not specified	not specified	[118]
gravity heat pipe (GHP)	self-rewetting fluids (SRWF)	copper	[48]
pulsating heat pipe (PHP)	self-rewetting nanofluids (SRNF)	copper	[132]
pulsating heat pipe (PHP)	de-ionized water PbSH_2_O, AuH_2_O, and GrapheneH_2_O nanofluids	copper	[135]
concentric annular heat pipe (CAHP)	distilled water	container, screen mesh wick—stainless steel Fins—copper	[105]
circular screen mesh wick heat pipe	water-based Al_2_O_3_ nanofluid	copper	[120]
sintered wick heat pipe (SWHP) and mesh wick heat pipe (MWHP)	de-ionized water CuO nanofluid	copper	[126,127]

**Table 5:** Reviews topics in the field of heat pipes research.

Year	Author	Title of Review	Sources	Highlights
2014	Amir Faghri	Heat pipes: Review, opportunities and challenges [138]	259	heat pipes in electronic cooling and energy sectors; flexibility of heat pipes and their ability to operate without external power; importance of numerical modeling and experimental simulations in enhancing understanding and performance of heat pipes.
2018	Y. Qu et al.	A review of thermal performance in multiple evaporators loop heat pipe [139]	68	investigated the development status of Multiple Evaporators Loop Heat Pipes (ME-LHP) focusing on system design, mathematical models, and operational performance; key factors influencing ME-LHP performance include the ratio of component volumes, working fluid mass, and operational temperature control.
2019	Mehdi Khiadani et al.	Theoretical modelling approaches of heat pipe solar collectors in solar systems: A comprehensive review [140]	114	theoretical modeling approaches of heat pipe solar collectors (HPSCs), highlighting their operational principles, advantages, and disadvantages; various mathematical models, including steady state and dynamic models, and discusses their applications in improving HPSC efficiency.
2021	Zhangyuan Wang et al.	A comprehensive review on the application of nanofluid in heat pipe based on the machine learning: Theory, application and prediction [141]	163	application of nanofluids in heat pipes, emphasizing the importance of viscosity, thermal conductivity, and stability in enhancing heat transfer performance; role of machine learning in modeling the thermal properties of nanofluids and identifies challenges such as uncertainties in thermal conductivity and viscosity, and limitations in predictive models; machine learning, particularly ANNs (Artificial Neural Network), can provide high prediction accuracy for nanofluid properties when combined with optimization algorithms.
2021	Cole Mueller et al.	A review of heat-pipe modeling and simulation approaches in nuclear systems design and analysis [142]	66	the historical context and evolution of heat-pipes, highlighting their transition from electronic systems to advanced nuclear applications; efficient approach for accurate heat-pipe modeling in multi-physics simulations, reducing computational time.
2023	Ishak Hashim et al.	Convection heat transfer in enclosures with inner bodies: A review on single and two-phase nanofluid models [143]	189	significant number of studies on natural and mixed convection in cavities with solid inner bodies, emphasizing the importance of geometry in heat exchange simulations; most research has utilized single-phase models due to the high numerical costs associated with two-phase models.
2023	June Kee Min et al.	A detailed review of pulsating heat pipe correlations and recent advances using Artificial Neural Network for improved performance prediction [144]	185	mathematical models for estimating the thermal performance of pulsating heat pipes (PHPs) and suggests improvements for prediction accuracy; the need for future studies to explore synergies between semi-empirical correlations and artificial neural networks (ANN) to enhance prediction capabilities.
2023	Mudasar Zafar et al.	Recent Development and Future Prospective of Tiwari and Das Mathematical Model in Nanofluid Flow for Different Geometries: A Review [145]	105	Tiwari and Das mathematical model for nanofluid flow, emphasizing its significance in enhancing heat transfer rates through various geometries; importance of accurate mathematical modeling and the effects of cavity shapes and thermal boundary conditions on fluid dynamics; copper nanoparticles are noted to provide the highest heat transmission rates, indicating their effectiveness in improving heat transfer performance.
2025	Mohammad Ghalambaz et al.	A review of technology, applications, and future perspectives of thermosyphons in permafrost regions [146]	236	thermosyphons play a vital role in thermal management, particularly in permafrost regions, by providing an efficient, passive heat transfer solution essential for infrastructure stability in cold climates; thermosyphons significantly improve soil stability and prevent thaw-induced damage, with innovative designs like L-shaped configurations showing enhanced cooling performance.

**Table 6:** Types of heat pipes and its applications. Shapes influence.

Shape	Applications
cylinder	Common cases such as normal size electronics, climate devices, industry heat systems
flat	Microelectronic devices, LED-lights
flexible	Spacecraft, cooling of medical devices, some uncommon industry systems with specific geometry
other	Unique devices and systems

**Table 7:** Types of heat pipes and its applications. Type of working fluid influence.

Working Temperature, K	Fluids	Applications
4–200	Helium, argon, krypton, oxygen	Spacecraft [151,152], medical devices [153]
200–550	Water, ammonia, acetone, the Freon compounds	Electronics and microelectronics cooling [154,155], permafrost cooling [156,157]
450–750	Mercury, sulphur, Thermex compound, Dowtherm-A compaund	Combustion engines and automotive industry [158,159], solar and geothermal systems [160,161], cooking [162,163]
>750	Sodium, lithium, cesium, silver, indium, sodium-potassium compound (NaK)	Nuclear power conversion [164,165], aerospace engineering [166]
